# Night shift work and risk of total and site-specific cancer: results from a prospective cohort study among Chinese men

**DOI:** 10.5271/sjweh.4290

**Published:** 2026-07-01

**Authors:** Qiu-Ming Shen, Zhuo-Ying Li, Yu-Ting Tan, Li-Feng Gao, Junior College,,, Da-Ke Liu, Hong-Lan Li, Wan-Shui Yang, Yong-Bing Xiang

**Affiliations:** 1 State Key Laboratory of Systems Medicine for Cancer, Shanghai Cancer Institute, Renji Hospital, Shanghai Jiao Tong University School of Medicine, Shanghai, China.; 2 Department of Epidemiology, Shanghai Cancer Institute, Shanghai, China.; 3 School of Public Health, Anhui Medical University, Hefei, China.

**Keywords:** epidemiology, night work, shift worker, cancer, cohort study

## Abstract

**Objective:**

Epidemiological evidence on the association between night shift work and cancer risk remains limited and inconsistent. This study aimed to systematically investigate this association among Chinese men.

**Methods:**

This population-based prospective cohort study included 61 078 men from the Shanghai Men’s Health Study. Detailed information on night shift work was collected at baseline using a structured questionnaire. Cox regression model was used to estimate hazard ratios (HR) and 95% confidence intervals (CI) for total cancer and ten major site-specific cancers. Restricted cubic spline functions were used to characterize the dose–response associations for key metrics. Further analysis was conducted with lag periods of 5, 10, 15 and 20 years, and potential effect modification by lifestyle factors was tested.

**Results:**

During a median follow-up period of 16.1 years, 8202 incident cancer cases were identified. Participants with 11–20 years of cumulative night shift work had a higher risk of pancreatic cancer compared with never-shift workers (HR 1.59, 95% CI 1.09–2.31). This association persisted across all lag periods tested, peaking at a 15-year lag (HR 1.82, 95% CI 1.25–2.64). No significant associations were found between night shift work metrics, including night shift work experience, starting age, cumulative duration, and frequency, and the risk of total and other major site-specific cancers. No evidence of effect modification by lifestyle factors was observed.

**Conclusions:**

Night shift work was not associated with the risk of overall or some common cancers among Chinese men. However, an increased risk of pancreatic cancer was associated with intermediate-to-long-term night shift work.

Night shift work, characterized by work conducted during the conventional sleeping hours of the general population, is a prevalent occupational exposure worldwide. According to statistics from the International Labor Organization, 5.7–25% of the global workforce engages in night shift work (1). In China, about 17.5% of workers report working night shifts at least once a month (2). This widespread phenomenon has garnered increasing attention as a potential risk factor for a spectrum of health problems, including metabolic syndrome, diabetes, mental and autoimmune disorders, cardiovascular diseases, and cancer (3).

The International Agency for Research on Cancer (IARC) has classified night shift work as “probably carcinogenic to humans (Group 2A)” based on sufficient evidence from animal studies and limited evidence in humans (4). To date, the epidemiological findings on the association between night shift work and cancer risk remain inconclusive. Among the most frequently investigated cancer sites – namely the breast, prostate, colon, and rectum – studies have reported both positive and null associations (5–9). For instance, while results from the Nurses’ Health Study II (10) and Swedish Twin registry (11) reported a positive association between night shift work and breast cancer risk, other large prospective studies, such as the Million Women Study, EPIC-Oxford and UK Biobank observed no clear association (12). Such inconsistency underscores the difficulty of drawing definitive conclusions from existing evidence and highlights a critical need for further high-quality prospective cohort studies. Besides, the evidentiary value of existing cohort studies is often constrained by methodological limitations beyond their design. A key shortcoming is weak exposure assessment, which limits precise estimation (13). Some studies observed an increased risk of breast cancer was only evident among long-term night shift workers (10, 11), suggesting that crude binary exposure measures may obscure important associations. This underscores the need for cohort studies that collect detailed metrics on night shift work – such as cumulative duration and frequency of exposure – to enable more comprehensive risk assessment. Furthermore, available evidence remains disproportionately focused on the aforementioned cancer types. The potential associations between night shift work and other common cancers, including lung, liver, and pancreas have scarcely been investigated, representing a significant gap in our understanding of the full carcinogenic potential of circadian disruption.

In China, epidemiological studies on the association between night shift work and cancer are limited. Most existing studies are case–control in design, and evidence from prospective cohort studies is notably scarce (14–20). Utilizing data from a large-scale, population-based prospective cohort, this study aims to systematically investigate the association between night shift work and cancer risk in the Chinese population. We examined various night shift work metrics, including ever exposure, age at starting, cumulative duration, and frequency, in relation to the risk of overall cancer and ten major site-specific cancers. We hypothesize that night shift work is associated with a higher risk of cancer. This study seeks to provide robust and detailed evidence that clarifies the impact of this common occupational exposure on cancer risk.

## Methods

### Study population

Participants included in this study were drawn from the Shanghai Men’s Health Study (SMHS), an ongoing, population-based prospective cohort that enrolled 61 469 men aged 40–74 years old from urban Shanghai between January 2002 and July 2006. The design and methodology of the cohort have been described previously (21). At baseline, all participants completed a structured questionnaire, which asked about demographic characteristics, lifestyle factors, dietary habits, medical history, and other relevant information. Anthropometric measurements were also obtained at baseline.

Exclusion criteria were applied as follows: (i) cancer at baseline; (ii) loss to follow-up shortly after the baseline survey; (iii) diagnosis of cancer in situ during follow-up; (iv) death from cancer with no cancer type or diagnosis date; (v) a cancer diagnosis that could not be confirmed; and (vi) without information about ever exposed to night shift work (supplementary material www.sjweh.fi/article/4290, figure S1). Altogether 61 078 men were retained in the current study.

### Assessment of night shift work

At the baseline survey, all participants were asked about any history of employment involving night shift work, defined as starting work after 22:00 hours ≥3 times a month for over one year. For participants who had night shift work experience, detailed information was collected regarding the years they started and finished working night shifts, the cumulative duration of night shift work, and the average times of night shifts they worked per month. The main night shift metrics that this study focused on included night shift work experience, age at starting night shift work, cumulative duration of night shift work, and frequency of night shifts.

### Covariates

Specially trained investigators collected information on age, education, income level, smoking status and alcohol consumption using a structured questionnaire in face-to-face interviews. Smoking was defined as ever smoking ≥1 cigarette per day for >6 months continuously. Alcohol consumption was defined as ever consuming alcohol ≥3 times per week for >6 months continuously. Body mass index (BMI) was calculated as weight in kilograms divided by height in meters squared, and was categorized according to the Chinese classification (22) into underweight (<18.5 kg/m^2^), normal (18.5–23.9 kg/m^2^), overweight (24–27.9 kg/m^2^) and obese (≥28.0 kg/m^2^). Physical activity was assessed using a semi-quantitative physical activity questionnaire (PAQ), and total activity was computed by summing metabolic equivalent task (MET) hours per week across all activities. Our previous study demonstrated that PAQ is a reproducible and valid tool for assessing physical activity (23). An 81-item semi-quantitative food-frequency questionnaire (FFQ), which covered approximately 88.8% of commonly consumed foods in urban Shanghai during the baseline years was used to assess usual food intake. Participants reported the frequency they consumed the food or food groups over the past 12 months, as well as the average amount of consumption in liangs (50 grams/liang) per unit of time. The FFQ has been proven to have substantial reproducibility and validity (24). Daily dietary intakes were energy-adjusted using the density method and standardized to 2000 kcal. We adopted the Chinese Food Pagoda score (CHFP) score to assess adherence to the Chinese dietary guidelines (25).

Possible covariates were considered in multivariable models, including age at baseline (continuous), education (elementary school or below/ middle school/ high school/ high profession, college or above), income level (<500, 500–999, 1000–1999, and ≥2000 yuan per month), BMI (underweight/normal/overweight/obese), smoking (ever/never), alcohol consumption (ever/never), physical activity (MET hour/week, quartiles) and CHFP (quartiles). Due to the low proportion of missing data in covariates (maximum missing frequency across all variables <1.3%), missing values were imputed using medians for continuous variables and modes for categorical variables.

### Assessment of outcomes

Since the baseline survey, the SMHS participants have been followed through a combination of active and passive methods. Active follow-up was conducted every 3–4 years through in-person interviews to update health status information. Passive follow-up was performed through annual record linkage to the Shanghai Cancer Registry for cancer incidence and the Shanghai Vital Statistics Registry for mortality data. Trained staff matched cohort members to registry data using unique identification numbers. Follow-up began at the date of baseline interview and continued until the date of cancer diagnosis, death, or 31 December 2020, whichever occurred first.

All diagnoses were coded according to the 9^th^ revision of the International Classification of Diseases (ICD-9). The outcomes in this study comprised the first diagnosis of all malignant cancers (ICD-9: 140–195, 200–208) and the following 10 major site-specific malignancies: lung (ICD-9: 162), colorectum (ICD-9:153, 154), liver (ICD-9: 155), stomach (ICD-9: 151), thyroid (ICD-9: 193), esophagus (ICD-9: 150), prostate (ICD-9: 185), bladder (ICD-9: 188), pancreas (ICD-9: 157) and kidney (ICD-9: 189).

### Statistical analysis

Baseline characteristics according to night shift experience were summarized as medians with interquartile ranges for continuous variables and counts with proportions for categorical variables. Mann-Whitney U test and χ^2^ test were performed to assess the difference between groups for continuous and categorical variables, respectively.

Detailed metrics pertaining to night shift work, including age at initiation, cumulative duration, and average monthly frequency, were visualized using box plots and compared between cancer and non-cancer groups using the Mann-Whitney U test. Cox proportional hazards regression models were used to evaluate the association between night shift metrics and cancer risk, with follow-up time as the underlying time scale. Follow-up time was calculated from the date of baseline survey until the date of outcome, death, loss to follow-up, or 31 December 2020, whichever occurred first. Proportional hazards assumption was tested by evaluating the correlation between Schoenfeld residuals and follow-up time, and no evidence of violation was observed. Results were presented as hazard ratios (HR) and 95% confidence intervals (CI), using participants without night shift experience as the reference group. Two models were fitted: model 1 was adjusted for age at baseline; model 2 was adjusted for age at baseline, education, income level, BMI, smoking, alcohol consumption, physical activity and CHFP score. Night shift metrics were first analyzed on categorical scale. For continuous variables, including cumulative duration of night shift work and frequency of night shifts, tests for linear trend were performed by entering these variables as continuous forms in the Cox regression models. To evaluate potential nonlinear associations, restricted cubic splines with three knots (10^th^, 50^th^, 90^th^ percentile) were applied to flexibly model the association between continuous night shift metrics and cancer risk among ever-night shift participants. Lagged analyses were conducted for night shift work duration by removing the most recent 5, 10, 15 and 20 years of night shift work exposure (fixing exposure to 5, 10, 15 and 20 years prior to baseline) to identify the long-term exposure contributed to cancer risk (26).

To examine the robustness of primary findings, sensitivity analyses were conducted. To minimize potential confounding from ongoing night shift work in the association between night shift work duration and cancer risk, we restricted the analysis to men who had ceased night shift work prior to baseline. For colorectal cancer and liver cancer, model 2 was further adjusted for a medical history of intestinal polyps and chronic hepatitis, respectively as they were strong risk factors for these cancers. However, these covariates were not retained in the final models because their inclusion neither changed the HR substantially nor improved the goodness of model fit (based on the Akaike information criterion, data not shown). To more accurately adjust smoking and alcohol consumption, categorical variables for the intensity of alcohol drinking (converted into ethanol intake per day) and the cumulative amount of cigarette smoking (defined as pack-year) were substituted for their binary forms in model 2, with each categorized as never-use or tertiles among users.

In addition, we performed prespecified exploratory analyses to evaluate whether lifestyle factors modified the associations between night shift metrics and cancer risk as recommended by IARC monographs on the identification of carcinogenic hazards to humans (4). We tested for interactions in the significant and robust associations by smoking, alcohol consumption, CHFP score, physical activity and BMI. Log-likelihood ratio tests were conducted to assess multiplicative interactions between night shift metrics and lifestyle factors by comparing the models with and without the cross-product terms. In another exploratory analysis, we examined the potential attenuation of cancer risk after cessation of night shift work. Ever night shift workers were categorized into three groups based on time since cessation: current workers, recent quitters (cessation ≤10 years) and long-term quitters (cessation >10 years). Each group was compared with never night shift workers using Cox regression models. We performed an additional analysis to evaluate the combined effects of duration and frequency on cancer risk. Participants were categorized into five mutually exclusive groups: never night shift (reference), short-term and low-intensity night shift (duration ≤10 years and frequency ≤8 nights/month); short-term and high-intensity night shift (duration ≤10 years and frequency >8 nights/month); long-term and low-intensity night shift (duration >10 years and frequency ≤8 nights/month); long-term and high-intensity night shift (duration >10 years and frequency >8 nights/month).

Statistical analyses were performed using SAS 9.4 (SAS Institute Inc, Cary, NC, USA). A two-tailed P-value <0.05 was considered statistically significant.

## Results

During a median follow-up of 16.1 years, a total of 8202 incident cancer cases were documented. The site-specific incidence numbers are presented in supplementary figure S2. Among 61 078 participants, 20 942 men had night shift work experience. Compared to those without night shift work experience, individuals with a history of night shift work were younger at baseline, had a greater proportion of lower education and income level, and were likely to report ever smoking, ever alcohol consumption and abnormal body weight. They also reported higher levels of total physical activity and lower CHFP score (table 1).

**Table 1:** Characteristics of the study participants in the Shanghai Men’s Health Study (N=61 078). [CHFP=Chinese Food Pagoda; MET=metabolic equivalent of task; IQR=interquartile range]

		Night shift workers (N=20 942)			Non-night shift workers (N=40 136)		P-value ^a^
		N (%)	Median (IQR)		N (%)	Median (IQR)	
Age (years)			52.46 (15.69)			53.45 (16.01)	<0.0001
Education							<0.0001
	Elementary school or below	1779 (8.49)			2256 (5.62)		
	Middle school	8387 (40.05)			11 839 (29.50)		
	High school	7882 (37.64)			14 675 (36.56)		
	High profession, college or above	2894 (13.82)			11 366 (28.32)		
Income level ^b^							<0.0001
	Low	3222 (15.39)			4468 (11.13)		
	Low to middle	10 039 (47.94)			16 008 (39.88)		
	Middle to high	6319 (30.17)			15 095 (37.61)		
	High	1362 (6.50)			4565 (11.37)		
Smoking							<0.0001
	Ever	15 548 (74.24)			26 989 (67.24)		
	Never	5394 (25.76)			13 147 (32.76)		
Alcohol consumption							<0.0001
	Ever	7883 (37.64)			12 706 (31.66)		
	Never	13 059 (62.36)			27 430 (68.34)		
Body mass index ^c^							0.0268
	Underweight	937 (4.47)			1640 (4.09)		
	Normal	10 422 (49.77)			20 157 (50.22)		
	Overweight	7804 (37.26)			15 105 (37.63)		
	Obese	1779 (8.49)			3234 (8.06)		
Total physical activity (MET hours/week)			58.02 (45.23)			52.03 (43.26)	<0.0001
CHFP score			30.28 (6.50)			31.19 (6.57)	<0.0001

^a^ Continuous variables were compared using Mann-Whitney U tests and categorical variables were compared using c^2^ test. ^b^ Personal income (yuan/month): low=<500; low to middle=500–999; middle to high=1000–1999; high=≥2,000. ^c^ BMI (kg/m^2^): underweight=<18.5; normal=18.5–<24.0; overweight=24.0–<28.0; obese=≥28.0.

Distributions of night shift work metrics between cancer and non-cancer groups are presented in supplementary figure S3. No statistically significant differences were observed between the cancer and non-cancer groups regarding age at starting night shift work or average monthly frequency of night shifts. A significant difference was found in the cumulative duration of night shift work (P=0.016).

In multivariable-adjusted models, none of the associations between night shift work experience and the risk of total or site-specific cancer reached statistical significance, except for kidney cancer (HR 0.77, 95% CI 0.60–0.99). However, since the upper confidence limit was close to null value, this association was not reliable for drawing substantive conclusions (table 2).

**Table 2:** Associations between night shift work experience and cancer risk among men. [HR=hazard ratio; CI=confidence interval.]

		Night shift work	N cases/participants	HR (95% CI) ^a^	HR (95% CI) ^b^
All cancers		Never	5375/40 136	1.00 (ref)	1.00 (ref)
		Ever	2827/20 942	1.05 (1.01–1.10)	1.01 (0.96–1.06)
Major cancer site					
	Lung	Never	1092/40 136	1.00 (ref)	1.00 (ref)
		Ever	645/20 942	1.18 (1.07–1.31)	1.05 (0.95–1.15)
	Colorectum	Never	900/40 136	1.00 (ref)	1.00 (ref)
		Ever	503/20 942	1.13 (1.01–1.26)	1.10 (0.99–1.23)
	Liver	Never	388/40 136	1.00 (ref)	1.00 (ref)
		Ever	204/20 942	1.04 (0.88–1.23)	0.96 (0.81–1.15)
	Stomach	Never	581/40 136	1.00 (ref)	1.00 (ref)
		Ever	321/20 942	1.12 (0.97–1.28)	1.04 (0.91–1.20)
	Thyroid	Never	96/40 136	1.00 (ref)	1.00 (ref)
		Ever	43/20 942	0.84 (0.59–1.21)	0.92 (0.64–1.33)
	Esophagus	Never	121/40 136	1.00 (ref)	1.00 (ref)
		Ever	70/20 942	1.16 (0.86–1.55)	0.90 (0.67–1.21)
	Prostate ^c^	Never	618/39 950	1.00 (ref)	1.00 (ref)
		Ever	246/20 851	0.82 (0.71–0.95)	0.89 (0.76–1.03)
	Bladder	Never	224/40 136	1.00 (ref)	1.00 (ref)
		Ever	102/20 942	0.92 (0.73–1.17)	0.92 (0.73–1.17)
	Pancreas	Never	229/40 136	1.00 (ref)	1.00 (ref)
		Ever	117/20 942	1.04 (0.83–1.29)	1.03 (0.82–1.29)
	Kidney	Never	235/40 136	1.00 (ref)	1.00 (ref)
		Ever	87/20 942	0.73 (0.57–0.93)	0.77 (0.60–0.99)

^a^ Adjusted for age at baseline. ^b^ Adjusted for age at baseline, education, income, smoking, alcohol consumption, CHFP score, physical activity and body mass index. ^c^ An additional 277 participants with a history of prostatectomy at baseline were excluded.

Age at starting night shift work did not appear to be associated with the risk of cancer (table 3). Starting night shift work at an earlier age (≤30 years) only seems to be associated with kidney cancer risk (HR 0.73, 95% CI 0.55–0.98) in the multivariable adjusted model. However, the marginal significance did not guarantee a firm conclusion and should be interpreted with caution.

**Table 3:** Associations between age at starting night shift work and cancer risk among men. [HR=hazard ratio; CI=confidence interval.]

		Age at starting night shift work	N cases/participants	HR (95% CI) ^a^	HR (95% CI) ^b^
All cancers		None	5375/40 135	1.00 (ref)	1.00 (ref)
		≤30 years old	2022/14 746	1.05 (1.00–1.11)	1.01 (0.96–1.07)
		31–40 years old	367/3003	1.05 (0.94–1.16)	1.00 (0.90–1.11)
		>40 years old	438/3188	1.08 (0.98–1.19)	1.00 (0.91–1.10)
Major cancer site					
	Lung	None	1092/40 135	1.00 (ref)	1.00 (ref)
		≤30 years old	458/14 746	1.18 (1.06–1.31)	1.06 (0.95–1.18)
		31–40 years old	77/3003	1.06 (0.84–1.34)	0.93 (0.74–1.17)
		>40 years old	110/3188	1.33 (1.10–1.62)	1.07 (0.88–1.31)
	Colorectum	None	900/40 135	1.00 (ref)	1.00 (ref)
		≤30 years old	361/14 746	1.13 (1.00–1.27)	1.11 (0.98–1.25)
		31–40 years old	60/3003	1.03 (0.79–1.34)	1.00 (0.77–1.31)
		>40 years old	82/3188	1.22 (0.97–1.53)	1.17 (0.93–1.48)
	Liver	None	388/40 135	1.00 (ref)	1.00 (ref)
		≤30 years old	136/14 746	0.98 (0.80–1.19)	0.91 (0.75–1.11)
		31–40 years old	30/3003	1.12 (0.77–1.63)	1.04 (0.71–1.51)
		>40 years old	38/3188	1.27 (0.91–1.77)	1.13 (0.80–1.58)
	Stomach	None	581/40 135	1.00 (ref)	1.00 (ref)
		≤30 years old	234/14 746	1.13 (0.97–1.32)	1.07 (0.91–1.24)
		31–40 years old	50/3003	1.35 (1.01–1.80)	1.25 (0.94–1.68)
		>40 years old	37/3188	0.86 (0.61–1.19)	0.76 (0.54–1.06)
	Thyroid	None	96/40 135	1.00 (ref)	1.00 (ref)
		≤30 years old	33/14746	0.93 (0.62–1.37)	1.00 (0.67–1.50)
		31–40 years old	5/3003	0.63 (0.25–1.54)	0.70 (0.28–1.72)
		>40 years old	5/3188	0.67 (0.27–1.65)	0.76 (0.31–1.90)
	Esophagus	None	121/40 135	1.00 (ref)	1.00 (ref)
		≤30 years old	47/14746	1.09 (0.78–1.52)	0.87 (0.62–1.23)
		31–40 years old	8/3003	0.99 (0.49–2.03)	0.76 (0.37–1.56)
		>40 years old	15/3188	1.63 (0.95–2.79)	1.10 (0.64–1.90)
	Prostate ^c^	None	618/39 949	1.00 (ref)	1.00 (ref)
		≤30 years old	184/14 680	0.84 (0.71–0.99)	0.90 (0.76–1.06)
		31–40 years old	24/2994	0.65 (0.43–0.97)	0.69 (0.46–1.04)
		>40 years old	38/3172	0.89 (0.64–1.23)	1.02 (0.73–1.42)
	Bladder	None	224/40 135	1.00 (ref)	1.00 (ref)
		≤30 years old	77/14746	0.97 (0.75–1.25)	0.97 (0.74–1.26)
		31–40 years old	14/3003	0.97 (0.57–1.67)	0.96 (0.56–1.65)
		>40 years old	11/3188	0.66 (0.36–1.21)	0.67 (0.36–1.23)
	Pancreas	None	229/40 135	1.00 (ref)	1.00 (ref)
		≤30 years old	98/14 746	1.13 (0.89–1.44)	1.12 (0.88–1.44)
		31–40 years old	11/3003	0.75 (0.41–1.37)	0.74 (0.40–1.36)
		>40 years old	14/3188	0.82 (0.48–1.42)	0.81 (0.47–1.39)
	Kidney	None	235/40 135	1.00 (ref)	1.00 (ref)
		≤30 years old	59/14 746	0.70 (0.52–0.93)	0.73 (0.55–0.98)
		31–40 years old	18/3003	1.08 (0.67–1.74)	1.16 (0.71–1.87)
		>40 years old	10/3188	0.54 (0.29–1.03)	0.59 (0.31–1.12)

^a^ Adjusted for age at baseline. ^b^ Adjusted for age at baseline, education, income, smoking, alcohol consumption, Chinese food pagoda score, physical activity and body mass index. ^c^ An additional 277 participants with a history of prostatectomy at baseline were excluded.

**Figure 1:**
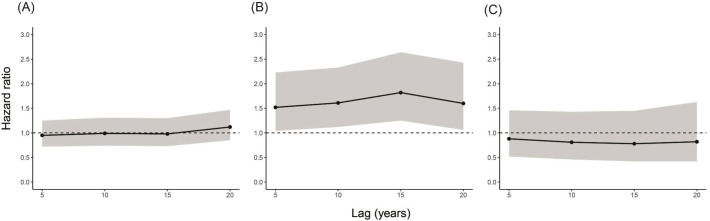
Associations between cumulative duration of night shift work and pancreatic cancer risk across 5-, 10-, 15- and 20-year lag periods. Hazard ratios and 95% confidence intervals are shown for (A) ≤10, (B) 11–20, and (C) >20 years of cumulative duration, using never night shift participants as the reference group.

Analysis of cumulative duration of night shift work revealed a positive association between 11–20 years of night shift work and pancreatic cancer risk (HR 1.59, 95% CI 1.09–2.31) after multivariable adjustments for potential confounders (table 4). No significant linear trends were observed between cumulative duration of night shift work and all cancers combined or major site-specific cancers in the multivariable-adjusted models (all P_trend_ >0.05). The exposure–hazard curve did not indicate any nonlinear associations (all P_nonlinear_ >0.05) (table 4). In lagged analyses, the association between 11–20 years of night shift work and pancreatic cancer risk remained statistically significant across all lag periods examined. The HR exhibited a generally increasing trend with longer lag intervals (5 years: 1.52; 10 years: 1.61; 15 years: 1.82), followed by slight attenuation in the 20-year lagged analysis, though the association remained significant (figure 1, supplementary table S1).

**Table 4:** Associations between cumulative duration of night shift work and cancer risk in men. [HR=hazard ratio; CI=confidence interval.]

		Cumulative duration of night shift work	N cases/participants	HR (95% CI) ^a^	HR (95% CI) ^b^	P_trend_^c^	P_nonlinear_^d^
All cancers		None	5375/40 135	1.00 (ref)	1.00 (ref)	0.4020	0.6083
		≤10 years	1827/13 879	1.04 (0.98–1.10)	1.01 (0.95–1.06)		
		11–20 years	510/3959	1.07 (0.97–1.17)	1.01 (0.92–1.11)		
		>20 years	490/3099	1.11 (1.01–1.21)	1.04 (0.95–1.14)		
Major cancer site							
	Lung	None	1092/40 135	1.00 (ref)	1.00 (ref)	0.5674	0.6969
		≤10 years	361/11 493	1.16 (1.04–1.30)	1.05 (0.94–1.17)		
		11–20 years	116/3959	1.19 (0.98–1.44)	1.02 (0.84–1.24)		
		>20 years	112/3099	1.27 (1.05–1.55)	1.06 (0.87–1.30)		
	Colorectum	None	900/40 135	1.00 (ref)	1.00 (ref)	0.1063	0.3405
		≤10 years	292/11 493	1.11 (0.98–1.26)	1.09 (0.96–1.24)		
		11–20 years	84/3959	1.06 (0.85–1.33)	1.03 (0.82–1.29)		
		>20 years	94/3099	1.26 (1.02–1.56)	1.22 (0.98–1.52)		
	Liver	None	388/40 135	1.00 (ref)	1.00 (ref)	0.6038	0.7515
		≤10 years	104/11 493	1.03 (0.84–1.25)	0.97 (0.79–1.18)		
		11–20 years	37/3959	1.03 (0.74–1.44)	0.94 (0.67–1.32)		
		>20 years	34/3099	1.11 (0.78–1.58)	0.98 (0.69–1.41)		
	Stomach	None	581/40 135	1.00 (ref)	1.00 (ref)	0.5680	0.7529
		≤10 years	191/11 493	1.12 (0.96–1.31)	1.06 (0.90–1.24)		
		11–20 years	63/3959	1.25 (0.96–1.62)	1.14 (0.88–1.48)		
		>20 years	48/3099	0.98 (0.73–1.31)	0.89 (0.66–1.20)		
	Thyroid	None	96/40 135	1.00 (ref)	1.00 (ref)	0.6745	0.266
		≤10 years	25/11 493	0.84 (0.55–1.27)	0.91 (0.60–1.38)		
		11–20 years	5/3959	0.50 (0.20–1.22)	0.56 (0.23–1.37)		
		>20 years	9/3099	1.40 (0.71–2.79)	1.62 (0.80–3.25)		
	Esophagus	None	121/40 135	1.00 (ref)	1.00 (ref)	0.3010	0.1448
		≤10 years	28/11 493	0.95 (0.66–1.37)	0.78 (0.54–1.12)		
		11–20 years	12/3959	1.10 (0.61–1.99)	0.83 (0.46–1.51)		
		>20 years	20/3099	2.04 (1.27–3.29)	1.40 (0.86–2.28)		
	Prostatee	None	618/39 949	1.00 (ref)	1.00 (ref)	0.4313	0.9342
		≤10 years	163/13 820	0.84 (0.71–1.00)	0.89 (0.75–1.06)		
		11–20 years	36/3943	0.71 (0.51–1.00)	0.78 (0.56–1.10)		
		>20 years	47/3083	0.85 (0.63–1.14)	0.96 (0.71–1.30)		
	Bladder	None	224/40 135	1.00 (ref)	1.00 (ref)	0.3487	0.6238
		≤10 years	60/11 493	0.94 (0.72–1.23)	0.94 (0.71–1.23)		
		11–20 years	20/3959	1.03 (0.65–1.62)	1.01 (0.64–1.61)		
		>20 years	14/3099	0.75 (0.44–1.29)	0.77 (0.44–1.32)		
	Pancreas	None	229/40 135	1.00 (ref)	1.00 (ref)	0.7441	0.1411
		≤10 years	64/11 493	0.95 (0.73–1.24)	0.94 (0.72–1.24)		
		11–20 years	32/3959	1.61 (1.11–2.34)	1.59 (1.09–2.31)		
		>20 years	15/3099	0.78 (0.46–1.32)	0.77 (0.46–1.31)		
	Kidney	None	235/40 135	1.00 (ref)	1.00 (ref)	0.6651	0.8635
		≤10 years	50/11 493	0.70 (0.52–0.94)	0.74 (0.55–1.00)		
		11–20 years	14/3959	0.63 (0.37–1.08)	0.68 (0.39–1.17)		
		>20 years	17/3099	0.95 (0.58–1.55)	1.02 (0.62–1.68)		
^a^ Adjusted for age at baseline. ^b^ Adjusted for age at baseline, education, income, smoking, alcohol consumption, Chinese food pagoda score, physical activity and body mass index. ^c^ Modeled as a continuous variable to test for linear trend. ^d^ Nonlinear associations were tested among participants with a history of night shift work. ^e^ An additional 277 participants with a history of prostatectomy at baseline were excluded.							

Although the age-adjusted model suggested that frequency of night shifts was associated with risk for total and some site-specific cancers, multivariable adjustment attenuated this association to non-significant (table 5). No significant linear trends (all P_trend_ >0.05) or non-linear associations (all P_nonlinear_ >0.05) were observed between shift frequency and cancer risks after multivariable adjustment (table 5).

**Table 5:** Associations between frequency of night shifts and cancer risk in men. [HR=hazard ratio; CI=confidence interval.]

		Average monthly frequency of night shifts	N cases/participants	HR (95% CI) ^a^	HR (95% CI) ^b^	P_trend_^c^	P_nonlinear_^d^
All cancers		None	5375/40 135	1.00 (ref)	1.00 (ref)	0.2522	0.5089
		≤8 nights/month	1900/14 390	1.02 (0.97–1.08)	0.98 (0.93–1.04)		
		>8 nights/month	927/6548	1.13 (1.05–1.21)	1.06 (0.99–1.14)		
Major cancer site							
	Lung	None	1092/40 135	1.00 (ref)	1.00 (ref)	0.0798	0.0602
		≤8 nights/month	422/14 390	1.12 (1.00–1.26)	1.00 (0.89–1.12)		
		>8 nights/month	223/6548	1.33 (1.15–1.53)	1.14 (0.98–1.31)		
	Colorectum	None	900/40 135	1.00 (ref)	1.00 (ref)	0.1526	0.9845
		≤8 nights/month	340/14 390	1.10 (0.97–1.25)	1.08 (0.95–1.23)		
		>8 nights/month	163/6548	1.19 (1.01–1.41)	1.15 (0.97–1.37)		
	Liver	None	388/40 135	1.00 (ref)	1.00 (ref)	0.7409	0.3739
		≤8 nights/month	136/14 390	1.00 (0.83–1.22)	0.93 (0.77–1.14)		
		>8 nights/month	68/6548	1.12 (0.86–1.45)	1.03 (0.79–1.33)		
	Stomach	None	581/40 135	1.00 (ref)	1.00 (ref)	0.3592	0.1091
		≤8 nights/month	214/14 390	1.07 (0.92–1.26)	1.01 (0.86–1.18)		
		>8 nights/month	107/6548	1.22 (0.99–1.50)	1.12 (0.91–1.38)		
	Thyroid	None	96/40 135	1.00 (ref)	1.00 (ref)	0.9827	0.4992
		≤8 nights/month	27/14 390	0.77 (0.51–1.19)	0.85 (0.55–1.31)		
		>8 nights/month	16/6548	0.98 (0.58–1.67)	1.08 (0.63–1.84)		
	Esophagus	None	121/40 135	1.00 (ref)	1.00 (ref)	0.7977	0.5329
		≤8 nights/month	43/14 390	1.03 (0.73–1.46)	0.82 (0.57–1.16)		
		>8 nights/month	27/6548	1.45 (0.95–2.20)	1.07 (0.70–1.63)		
	Prostatee	None	618/39 949	1.00 (ref)	1.00 (ref)	0.1514	0.9132
		≤8 nights/month	163/14 318	0.78 (0.66–0.93)	0.84 (0.70–1.00)		
		>8 nights/month	83/6529	0.92 (0.73–1.15)	1.00 (0.79–1.26)		
	Bladder	None	224/40 135	1.00 (ref)	1.00 (ref)	0.9944	0.2766
		≤8 nights/month	70/14 390	0.91 (0.70–1.19)	0.91 (0.70–1.20)		
		>8 nights/month	32/6548	0.95 (0.65–1.37)	0.94 (0.65–1.37)		
	Pancreas	None	229/40 135	1.00 (ref)	1.00 (ref)	0.8673	0.2356
		≤8 nights/month	81/14 390	1.03 (0.80–1.33)	1.03 (0.79–1.33)		
		>8 nights/month	36/6548	1.04 (0.73–1.48)	1.03 (0.72–1.46)		
	Kidney	None	235/40 135	1.00 (ref)	1.00 (ref)	0.1026	0.2262
		≤8 nights/month	61/14 390	0.74 (0.56–0.98)	0.79 (0.59–1.05)		
		>8 nights/month	26/6548	0.69 (0.46–1.04)	0.73 (0.49–1.10)		
^a^ Adjusted for age at baseline. ^b^ Adjusted for age at baseline, education, income, smoking, alcohol consumption, Chinese food pagoda score, physical activity and body mass index. ^c^ Modeled as a continuous variable to test for linear trend. ^d^ Nonlinear associations were tested among participants with a history of night shift work. ^e^ An additional 277 participants with a history of prostatectomy at baseline were excluded.							

The results of the sensitivity analyses largely corroborated the primary findings (supplementary table S2–S7). Following a conservative approach that excluded associations which were non-significant, of marginal significance, or unrobust, only the association between cumulative duration of night shift work and pancreatic cancer risk was retained for assessment of effect modification. Log-likelihood ratio tests did not indicate interactions between cumulative duration of night shift work and any lifestyle factors examined (all P >0.05) (supplementary figure S4). In an exploratory analysis examining the potential attenuation of risk after cessation of night shift work, some site-specific associations between certain night shift metrics and cancer risk were recognized, although these associations were observed only in specific cessation subgroups rather than consistently across all cessation categories. These findings do not support a clear interpretation of risk attenuation after cessation of night shift work (supplementary table S8–S10). In analysis examining the combined effects of night shift work duration and frequency, no statistically significant associations were observed between any of the four exposure groups and total or major site-specific cancer risk (supplementary table S11).

## Discussion

In this large-scale prospective cohort study among Chinese men, we found an increased risk of pancreatic cancer for participants with 11–20 years of cumulative night shift work, compared to those who never engaged in night shift work. No significant associations were found for other night shift work metrics, including night shift work experience, age at starting, cumulative duration, or frequency in relation to the risk of overall cancer or other major site-specific cancers.

This study did not observe the significant associations between night shift work metrics and the risk of overall cancer or some common cancers. The null finding for overall cancer is consistent with previous studies conducted in Japanese (27) and German (28) populations. However, the results for certain common cancers appear to differ from the 2019 IARC evaluation, which concluded that positive associations have been observed between night shift work and cancers of the breast, prostate, colon, and rectum (4). The biological plausibility of these associations is supported by shared mechanisms, including melatonin disruption (29), alterations in clock gene expression (30), and impaired immune function (31), as well as cancer-specific pathways such as sex hormone signaling in breast (32) and prostate (33) cancers and gut microbiome disruption in colorectal cancer (34). Notably, evidence from prospective studies published after the 2019 IARC evaluation regarding these cancers remains inconsistent, with both positive and null associations reported for breast (5), prostate (6, 7), and colorectal cancers (8, 9). Population heterogeneity in the capacity of maintaining a normal circadian pattern (35) and inconsistent definitions of exposure (12) may be critical factors that explain inconsistent findings across studies. For cancer sites other than those specified by IARC, although positive associations for some night shift work metrics were observed in cancers of lung (36), esophagus (27), thyroid (37), bladder (38), among others, these risks were not consistently seen across the body of evidence (39–44). Overall, the existing evidence for these cancer outcomes remains preliminary and contradictory.

This study suggests that cumulative night shift work may be associated with an increased risk of pancreatic cancer, although a statistically significant association was observed only for intermediate-to-long-term exposure duration (11–20 years). Short-term exposure (≤10 years) may indicate a potential risk-accumulation phase where the exposure has not yet reached the threshold necessary to significantly alter cancer risk. Conversely, the non-significant association for long-term exposure (>20 years) may reflect the influence of the healthy worker effect (45). The significant association identified specifically for the 11–20 years exposure window potentially represents a critical etiologically relevant period for carcinogenesis. This duration may be sufficient for chronic circadian disruption, through mechanisms such as suppression of melatonin and dysregulation of clock genes, to cause cumulative damage to metabolic and cellular repair processes, thereby reaching a threshold that significantly promotes cancer development (46–48).

Our finding of a positive association between 11–20 years of cumulative night shift work and pancreatic cancer risk contributes to the mixed body of evidence on this topic, which includes both supportive and null results. A case–control study of men in Canada reported that ever performing night shift work was associated with a 127% higher risk of pancreatic cancer [odds ratio (OR) 2.27, 95% CI 1.24–4.15] (49). Suggestive elevations in risk – greater than twofold – were also observed for durations of 5–10 and >10 years of cumulative night shift work, with wide CI rendering these estimates statistically non-significant and imprecise (50). It is important to note that the available cohort evidence has generally been limited by a lack of detailed metrics of night shift work (such as cumulative duration) and has largely reported null associations with pancreatic cancer (27, 28, 50, 51). Collectively, the current epidemiological evidence remains inconclusive, highlighting the need for further prospective studies with refined night shift work assessment to clarify this association.

Despite the lack of epidemiological consensus, a biologically plausible link between night shift work and pancreatic cancer risk is supported by the melatonin hypothesis (29). This hypothesis establishes a mechanistic link between circadian disruption – particularly night shift work – and cancer risk by proposing that light at night suppresses nocturnal melatonin production, diminishing its anticancer properties. Melatonin protects against pancreatic carcinogenesis through multiple pathways (52): it confers protection against inflammatory damage and oxidative stress in the pancreas by enhancing antioxidant enzyme activity and scavenging reactive oxygen and nitrogen species; it also reduces endothelial cell proliferation and angiogenesis through inhibition of vascular endothelial growth factor; low levels of melatonin promote the expression of anti-apoptotic heat shock proteins, thereby blocking caspase-3 activation and inhibiting apoptosis. Collectively, disruption of melatonin signaling due to light at night compromises the protective network, potentially facilitating the biological processes of pancreatic cancer, including chronic inflammation, uncontrolled proliferation, and resistance to cell death. In addition to the melatonin pathway, night shift work has also been shown to disrupt the circadian rhythmicity of DNA repair genes and elevate DNA damage among humans, providing a direct genotoxic mechanism linking circadian disruption to carcinogenic risk (53). Besides, night shift work disrupts circadian rhythms in pancreatic islets, leading to impaired insulin secretion, insulin resistance, and glucose intolerance (54). This establishes a hyperglycemic metabolic environment that predisposes to type 2 diabetes mellitus, a well-established risk factor that significantly elevates the incidence of pancreatic cancer (55). Together, these mechanisms offer complementary biological rationales supporting a potential association between night shift work and pancreatic carcinogenesis.

The positive association between 11–20 years of night shift work and pancreatic cancer risk remained robust across all sensitivity analyses. Notably, this association exhibited a generally increasing trend with longer lag intervals, specifically over the 5-, 10-, and 15-year lag periods. Although slight attenuation was observed at the 20-year lag compared to the preceding increasing trend, the association remained statistically significant. These results are similar to those from a previously mentioned case–control study conducted among Canadian men, which reported a markedly elevated risk of pancreatic cancer associated with night shift work performed ≤20 years prior to diagnosis or interview (OR 3.81, 95% CI 1.75–8.28), along with a weaker, non-significant increase in risk for night work conducted >20 years prior to diagnosis or interview (OR 1.49, 95% CI 0.55–4.06) (49). Both studies suggest a lag effect of night shift work, indicating that past exposure dating back as far as 20 years prior to baseline or diagnosis may still contribute to cancer risk. In the present study, the strengthening association with earlier exposure windows, peaking at around 15-year lag, indicates that night shift work exerts a long-term effect on pancreatic cancer risk, with a prolonged induction or latency period of over a decade.

This prospective cohort study provided a comprehensive assessment of the association between night shift work and cancer risk, utilizing detailed shift work metrics and examining incidence across ten major cancer sites as well as all cancers combined in the Chinese men. The study extends previous research by incorporating underreported exposure details, investigating seldom-explored non-linear dose–response associations for key metrics, employing lagged analyses to account for long-term contribution of past exposure, and evaluating potential effect modification by lifestyle factors. Several limitations should be considered when interpreting our findings. First, night shift exposure assessments were derived exclusively from self-reported questionnaires at baseline, which was susceptible to recall bias and did not capture dynamic changes over time. This may have led to non-differential misclassification of exposure, which likely biases our effect estimates toward the null. Consequently, the true associations between night shift work and cancer risk in this population may be stronger than those observed. Future studies with repeated, longitudinal exposure assessments are warranted to better characterize long-term exposure patterns and their associations with cancer risk. Second, while genetic polymorphisms in circadian rhythm genes were known to modulate physiological responses to night shifts and influence cancer susceptibility (56), the lack of genetic data precluded the examination of such gene-environment interactions. Third, detailed occupational histories were not available, thereby preventing control for potential confounding from workplace carcinogens that frequently co-occur with night shift work in certain industries (57, 58). Finally, although we adjusted for a range of available confounders, residual confounding due to unmeasured or unknown factors cannot be ruled out, as is inherent in observational studies (59).

### Concluding remarks

In conclusion, this large-scale prospective cohort study of Chinese men found no significant association for night shift work metrics and the risk of overall cancer or some common cancers. But there is a significant association between intermediate-to-long-term (11–20 years) cumulative night shift work and an increased risk of pancreatic cancer. These results provided robust epidemiological findings from an understudied population and refined our understanding of the carcinogenic potential of this common occupational exposure.

## Supplementary material

Supplementary materials

## Protection of research participants

This study was carried out in accordance with Declaration of Helsinki. The Institutional Review Boards of the Shanghai Cancer Institute and Vanderbilt University approved the cohort protocol. The Renji Hospital Ethics Committee of Shanghai Jiao Tong University School of Medicine (KY2024-050-C) approved the study.
