# Supplementary material for: Night shift work and risk of total and site-specific cancer: results from a prospective cohort study among Chinese men
Source: Scand J Work Environ Health. 2026 Jun 26;52(4):391–402. doi: 10.5271/sjweh.4290 (PMC13344439; doi:10.5271/sjweh.4290)
Supplement: Supplementary materials [file SJWEH-52-391-S001.pdf]

Night shift work and risk of total and site-specific cancer: results from a prospective cohort study among Chinese men[1]

by Qiu-Ming Shen, PhD, Zhuo-Ying Li, PhD, Yu-Ting Tan, MD, MSc, Li-Feng Gao, Junior College, Da-Ke Liu, BA, Hong-Lan Li, MD, MPH, Wan-Shui Yang, PhD, Yong-Bing Xiang, MD, MSc [2]

1. Supplementary materials
2. Correspondence to: Yong-Bing Xiang, Department of Epidemiology & State Key Laboratory of Systems Medicine for Cancer, Shanghai Cancer Institute, Renji Hospital, Shanghai Jiao Tong University School of Medicine, Shanghai, 200032, P. R. China. [E-mail: ybxiang@shsci.org]. ORCID: 0000-0002-3840-9915

Table S1 Associations between cumulative duration of night shift work and cancer risk in 5-, 10-, 15- and 20-year lagged analyses
Table S2 Associations between cumulative duration of night shift work and cancer risk after excluding participants with ongoing night shift work at baseline
Table S3 Associations between night shift work and cancer risk with further adjustment for medical history in colorectal and liver cancer
Table S4 Associations between night shift work experience and cancer risk in men with more accurate adjustment for smoking and alcohol use
Table S5 Associations between age at starting night shift work and cancer risk in men with more accurate adjustment for smoking and alcohol use
Table S6 Associations between cumulative duration of night shift work and cancer risk in men with more accurate adjustment for smoking and alcohol use
Table S7 Associations between frequency of night shifts and the risk cancer risk in men with more accurate adjustment for smoking and alcohol use
Table S8 Associations between age at starting night shift work and cancer risk by cessation of night shift work
Table S9 Associations between cumulative duration of night shift work and cancer risk by cessation of night shift work
Table S10 Associations between frequency of night shifts and cancer risk by cessation of night shift work
Table S11 Associations between combinations of night shift work duration and frequency and cancer risk
Figure S1 Figure S1 Flowchart of study participants
Figure S2 Count and proportion of incident cancer cases by 10 major sites
Figure S3 Distribution of night shift work metrics between cancer and non-cancer groups
Figure S4 Stratified analysis of the association between cumulative duration of night shift work and pancreatic cancer risk according to lifestyle factors

Table S1 Associations between cumulative duration of night shift work and cancer risk in 5-, 10-, 15- and 20-year lagged analyses

Cumulative duration of night shift work (years)		5-year lag		10-year lag		15-year lag		20-year lag	
		No. of cases/No. of participants	HR (95%CI) [a]	No. of cases/No. of participants	HR (95%CI) [a]	No. of cases/No. of participants	HR (95%CI) [a]	No. of cases/No. of participants	HR (95%CI) [a]
All cancers	none	5553/41981	1.00 (ref)	5740/43602	1.00 (ref)	5872/44739	1.00 (ref)	6013/46123	1.00 (ref)
	≤10	1663/12242	1.01 (0.95-1.07)	1528/11107	1.00 (0.95-1.06)	1531/11456	1.00 (0.95-1.06)	1533/11369	1.02 (0.96-1.08)
	11-20	521/4109	1.02 (0.93-1.11)	548/4260	1.05 (0.96-1.14)	451/3199	1.02 (0.93-1.12)	391/2346	1.01 (0.91-1.12)
	>20	465/2740	1.05 (0.96-1.16)	386/2103	1.00 (0.90-1.11)	348/1678	1.04 (0.93-1.16)	265/1234	1.00 (0.88-1.14)
Major cancer site									
Lung	none	1142/41981	1.00 (ref)	1180/43602	1.00 (ref)	1210/44739	1.00 (ref)	1239/46123	1.00 (ref)
	≤10	372/12242	1.03 (0.92-1.16)	345/11107	1.06 (0.94-1.19)	347/11456	1.06 (0.94-1.19)	354/11369	1.10 (0.97-1.24)
	11-20	115/4109	0.99 (0.81-1.20)	129/4260	1.09 (0.91-1.31)	103/3199	1.05 (0.86-1.29)	87/2346	1.04 (0.84-1.30)
	>20	108/2740	1.09 (0.89-1.33)	83/2103	0.98 (0.78-1.23)	77/1678	1.05 (0.83-1.33)	57/1234	0.98 (0.75-1.30)
Colorectum	none	930/41981	1.00 (ref)	961/43602	1.00 (ref)	982/44739	1.00 (ref)	1008/46123	1.00 (ref)
	≤10	303/12242	1.12 (0.98-1.28)	281/11107	1.12 (0.98-1.28)	282/11456	1.12 (0.98-1.28)	281/11369	1.13 (0.99-1.29)
	11-20	84/4109	1.01 (0.81-1.27)	90/4260	1.06 (0.85-1.31)	74/3199	1.02 (0.81-1.30)	60/2346	0.93 (0.72-1.21)
	>20	86/2740	1.19 (0.95-1.49)	71/2103	1.12 (0.87-1.43)	65/1678	1.17 (0.90-1.52)	54/1234	1.23 (0.93-1.64)
Liver	none	408/41981	1.00 (ref)	427/43602	1.00 (ref)	441/44739	1.00 (ref)	453/46123	1.00 (ref)
	≤10	115/12242	0.93 (0.75-1.14)	101/11107	0.89 (0.71-1.10)	93/11456	0.80 (0.64-1.00)	97/11369	0.84 (0.68-1.05)
	11-20	31/4109	0.76 (0.53-1.10)	35/4260	0.83 (0.58-1.17)	32/3199	0.94 (0.65-1.35)	21/2346	0.76 (0.49-1.19)
	>20	38/2740	1.19 (0.85-1.68)	29/2103	1.09 (0.74-1.60)	26/1678	1.15 (0.76-1.73)	21/1234	1.20 (0.76-1.89)
Stomach	none	597/41981	1.00 (ref)	610/43602	1.00 (ref)	627/44739	1.00 (ref)	652/46123	1.00 (ref)
	≤10	196/12242	1.09 (0.93-1.28)	190/11107	1.16 (0.98-1.36)	186/11456	1.13 (0.96-1.33)	173/11369	1.05 (0.88-1.24)
	11-20	66/4109	1.19 (0.92-1.53)	62/4260	1.10 (0.85-1.44)	53/3199	1.09 (0.82-1.45)	51/2346	1.15 (0.87-1.54)
	>20	43/2740	0.85 (0.62-1.16)	40/2103	0.90 (0.65-1.25)	36/1678	0.92 (0.65-1.29)	26/1234	0.81 (0.54-1.22)
Thyroid	none	98/41981	1.00 (ref)	102/43602	1.00 (ref)	105/44739	1.00 (ref)	105/46123	1.00 (ref)
	≤10	27/12242	0.99 (0.65-1.53)	23/11107	0.94 (0.60-1.48)	23/11456	0.89 (0.57-1.41)	29/11369	1.19 (0.79-1.80)
	11-20	7/4109	0.76 (0.35-1.65)	10/4260	1.06 (0.55-2.05)	9/3199	1.40 (0.71-2.79)	3/2346	0.77 (0.24-2.45)
	>20	7/2740	1.62 (0.74-3.55)	4/2103	1.39 (0.50-3.87)	2/1678	1.01 (0.24-4.23)	2/1234	1.66 (0.39-7.15)

Esophagus	none	130/41981	1.00 (ref)	136/43602	1.00 (ref)	140/44739	1.00 (ref)	145/46123	1.00 (ref)
	≤10	29/12242	0.65 (0.43-0.97)	27/11107	0.67 (0.44-1.01)	27/11456	0.66 (0.44-1.00)	27/11369	0.67 (0.44-1.01)
	11-20	15/4109	0.99 (0.58-1.70)	15/4260	0.97 (0.57-1.66)	11/3199	0.88 (0.47-1.63)	10/2346	0.94 (0.49-1.79)
	>20	17/2740	1.24 (0.74-2.08)	13/2103	1.09 (0.61-1.96)	13/1678	1.25 (0.69-2.27)	9/1234	1.06 (0.53-2.14)
Prostate[b]	none	628/41794	1.00 (ref)	638/43415	1.00 (ref)	648/44550	1.00 (ref)	657/45930	1.00 (ref)
	≤10	149/12184	0.87 (0.73-1.04)	141/11049	0.88 (0.73-1.06)	133/11400	0.84 (0.69-1.01)	129/11315	0.83 (0.68-1.00)
	11-20	38/4095	0.83 (0.59-1.15)	36/4246	0.78 (0.56-1.09)	39/3184	0.92 (0.67-1.28)	45/2333	1.08 (0.80-1.46)
	>20	49/2722	1.04 (0.77-1.40)	49/2085	1.12 (0.83-1.52)	44/1661	1.10 (0.80-1.50)	33/1217	1.00 (0.70-1.44)
Bladder	none	229/41981	1.00 (ref)	233/43602	1.00 (ref)	240/44739	1.00 (ref)	245/46123	1.00 (ref)
	≤10	64/12242	0.97 (0.74-1.28)	62/11107	1.03 (0.77-1.36)	59/11456	0.97 (0.72-1.28)	59/11369	0.98 (0.73-1.30)
	11-20	19/4109	0.95 (0.60-1.53)	19/4260	0.94 (0.59-1.51)	16/3199	0.92 (0.55-1.53)	14/2346	0.90 (0.52-1.55)
	>20	14/2740	0.82 (0.47-1.42)	12/2103	0.81 (0.45-1.46)	11/1678	0.84 (0.45-1.55)	8/1234	0.76 (0.37-1.57)
Pancreas	none	235/41981	1.00 (ref)	239/43602	1.00 (ref)	243/44739	1.00 (ref)	245/46123	1.00 (ref)
	≤10	64/12242	0.95 (0.72-1.25)	61/11107	0.99 (0.74-1.31)	60/11456	0.98 (0.73-1.30)	67/11369	1.12 (0.85-1.47)
	11-20	31/4109	1.52 (1.04-2.23)	33/4260	1.61 (1.12-2.33)	32/3199	1.82 (1.25-2.64)	25/2346	1.60 (1.06-2.43)
	>20	16/2740	0.88 (0.52-1.46)	13/2103	0.81 (0.46-1.43)	11/1678	0.78 (0.42-1.45)	9/1234	0.82 (0.42-1.63)
Kidney	none	239/41981	1.00 (ref)	246/43602	1.00 (ref)	248/44739	1.00 (ref)	254/46123	1.00 (ref)
	≤10	52/12242	0.79 (0.58-1.07)	47/11107	0.78 (0.57-1.07)	50/11456	0.83 (0.61-1.12)	47/11369	0.79 (0.58-1.08)
	11-20	16/4109	0.77 (0.46-1.28)	17/4260	0.80 (0.49-1.31)	14/3199	0.85 (0.49-1.46)	15/2346	1.14 (0.67-1.93)
	>20	15/2740	1.03 (0.60-1.75)	12/2103	1.03 (0.57-1.87)	10/1678	1.07 (0.56-2.06)	6/1234	0.88 (0.38-2.03)

a. Adjusted for age at baseline, education, income, smoking, alcohol use, CHFP score, physical activity and BMI.

b. An additional 277 participants with a history of prostatectomy at baseline were excluded.

Table S2 Associations between cumulative duration of night shift work and cancer risk after excluding participants with ongoing night shift work at baseline

	Cumulative duration of night shift work	No. of cases/No. of participants	HR (95%CI) [a]	P_{trend} [b]	$P_{nonlinear}$ [c]
All cancers	None	5375/40135	1.00 (ref)	0.5975	0.3224
	≤10 years	1594/11493	1.01 (0.95-1.06)		
	11-20 years	442/3310	0.99 (0.90-1.10)		
	>20 years	403/2234	1.04 (0.93-1.15)		
Major cancer site					
Lung	None	1092/40135	1.00 (ref)	0.6784	0.9743
	≤10 years	361/11493	1.06 (0.94-1.19)		
	11-20 years	102/3310	1.02 (0.83-1.26)		
	>20 years	91/2234	1.06 (0.85-1.32)		
Colorectum	None	900/40135	1.00 (ref)	0.0869	0.1258
	≤10 years	292/11493	1.12 (0.98-1.28)		
	11-20 years	71/3310	0.98 (0.77-1.26)		
	>20 years	81/2234	1.28 (1.01-1.61)		
Liver	None	388/40135	1.00 (ref)	0.7169	0.5131
	≤10 years	104/11493	0.89 (0.72-1.11)		
	11-20 years	24/3310	0.71 (0.47-1.08)		
	>20 years	30/2234	1.10 (0.75-1.61)		
Stomach	None	581/40135	1.00 (ref)	0.6867	0.6499
	≤10 years	191/11493	1.10 (0.93-1.30)		
	11-20 years	57/3310	1.16 (0.88-1.52)		
	>20 years	41/2234	0.90 (0.65-1.25)		
Thyroid	None	96/40135	1.00 (ref)	0.9142	0.7761
	≤10 years	25/11493	0.97 (0.62-1.51)		
	11-20 years	5/3310	0.69 (0.28-1.71)		
	>20 years	4/2234	1.18 (0.42-3.27)		

Esophagus	None	121/40135	1.00 (ref)	0.5051	0.403
	≤10 years	28/11493	0.67 (0.44-1.02)		
	11-20 years	12/3310	0.95 (0.52-1.72)		
	>20 years	14/2234	1.15 (0.65-2.04)		
Prostate[d]	None	618/39949	1.00 (ref)	0.2395	0.8268
	≤10 years	150/11435	0.89 (0.75-1.07)		
	11-20 years	36/3294	0.84 (0.60-1.19)		
	>20 years	44/2218	1.01 (0.74-1.38)		
Bladder	None	224/40135	1.00 (ref)	0.7909	0.418
	≤10 years	60/11493	0.93 (0.70-1.24)		
	11-20 years	16/3310	0.90 (0.54-1.51)		
	>20 years	11/2234	0.70 (0.38-1.30)		
Pancreas	None	229/40135	1.00 (ref)	0.7434	0.1354
	≤10 years	64/11493	0.98 (0.74-1.30)		
	11-20 years	29/3310	1.61 (1.09-2.38)		
	>20 years	11/2234	0.68 (0.37-1.25)		
Kidney	None	235/40135	1.00 (ref)	0.5772	0.898
	≤10 years	50/11493	0.78 (0.58-1.07)		
	11-20 years	12/3310	0.68 (0.38-1.22)		
	>20 years	12/2234	0.98 (0.54-1.77)		

a. Adjusted for age at baseline, education, income, smoking, alcohol use, CHFP score, physical activity and BMI.

b. Modelled as a continuous variable to test for linear trend.

c. Nonlinear associations were tested among participants with a history of night shift work.

d. An additional 276 participants with a history of prostatectomy at baseline were excluded.

Table S3 Associations between night shift work and cancer risk with further adjustment for medical history in colorectal and liver cancer

	Colorectal cancer				Liver cancer			
	No. of cases/No. of participants	HR (95%CI) [a]	P_{trend} [c]	$P_{nonlinear}$ [d]	No. of cases/No. of participants	HR (95%CI) [b]	P_{trend} [c]	$P_{nonlinear}$ [d]
Night shift work								
never	900/40136	1.00 (ref)			388/40136	1.00 (ref)		
ever	503/20942	1.10 (0.99-1.23)			204/20942	0.96 (0.81-1.14)		
Age at starting night shift work								
none	900/40135	1.00 (ref)			388/40135	1.00 (ref)		
≤30 years old	361/14746	1.11 (0.98-1.25)			136/14746	0.90 (0.74-1.10)		
31-40 years old	60/3003	1.01 (0.77-1.31)			30/3003	0.99 (0.68-1.43)		
>40 years old	82/3188	1.17 (0.93-1.48)			38/3188	1.18 (0.84-1.66)		
Cumulative duration of night shift work								
none	900/40135	1.00 (ref)	0.1058	0.3421	388/40135	1.00 (ref)	0.4185	0.8156
≤10 years	292/11493	0.90 (0.74-1.10)			104/11493	0.94 (0.77-1.15)		
11-20 years	84/3959	0.99 (0.68-1.43)			37/3959	0.94 (0.67-1.32)		
>20 years	94/3099	1.18 (0.84-1.66)			34/3099	1.06 (0.74-1.51)		
Average monthly frequency of night shifts								
none	900/40135	1.00 (ref)	0.1524	0.9805	388/40135	1.00 (ref)	0.9037	0.4117
≤8 nights/month	340/14390	1.08 (0.95-1.23)			136/14390	0.93 (0.76-1.13)		
>8 nights/month	163/6548	1.15 (0.97-1.37)			68/6548	1.02 (0.78-1.32)		

a. Adjusted for age at baseline, education, income, smoking, alcohol use, CHFP score, physical activity, BMI and medical history of intestinal polyp.
b. Adjusted for age at baseline, education, income, smoking, alcohol use, CHFP score, physical activity, BMI and medical history of chronic hepatitis.
c. Modeled as a continuous variable to test for linear trend.
d. Nonlinear associations were tested among participants with a history of night shift work.

Table S4 Associations between night shift work experience and cancer risk in men with more accurate adjustment for smoking and alcohol use

	Night shift work experience	No. of cases/No. of participants	HR (95%CI) [a]
All cancers	Never	5375/40136	1.00 (ref)
	Ever	2827/20942	1.01 (0.96-1.06)
Major cancer site			
Lung	Never	1092/40136	1.00 (ref)
	Ever	645/20942	1.05 (0.95-1.15)
Colorectum	Never	900/40136	1.00 (ref)
	Ever	503/20942	1.10 (0.99-1.23)
Liver	Never	388/40136	1.00 (ref)
	Ever	204/20942	0.96 (0.81-1.15)
Stomach	Never	581/40136	1.00 (ref)
	Ever	321/20942	1.04 (0.91-1.20)
Thyroid	Never	96/40136	1.00 (ref)
	Ever	43/20942	0.92 (0.64-1.33)
Esophagus	Never	121/40136	1.00 (ref)
	Ever	70/20942	0.90 (0.67-1.21)
Prostate[b]	Never	618/39950	1.00 (ref)
	Ever	246/20851	0.89 (0.76-1.03)
Bladder	Never	224/40136	1.00 (ref)
	Ever	102/20942	0.92 (0.73-1.17)
Pancreas	Never	229/40136	1.00 (ref)
	Ever	117/20942	1.03 (0.82-1.29)
Kidney	Never	235/40136	1.00 (ref)
	Ever	87/20942	0.77 (0.60-0.99)

a. Adjusted for age at baseline, education, income, smoking, alcohol use, CHFP score, physical activity and BMI.
b. An additional 277 participants with a history of prostatectomy at baseline were excluded.

Table S5 Associations between age at starting night shift work and cancer risk in men with more accurate adjustment for smoking and alcohol use

	Age at starting night shift work	No. of cases/No. of participants	HR (95%CI) [a]
All cancers	None	5375/40135	1.00 (ref)
	≤30 years old	2022/14746	1.01 (0.96-1.07)
	31-40 years old	367/3003	1.00 (0.90-1.11)
	>40 years old	438/3188	1.00 (0.91-1.10)
Major cancer site			
Lung	None	1092/40135	1.00 (ref)
	≤30 years old	458/14746	1.06 (0.95-1.18)
	31-40 years old	77/3003	0.93 (0.74-1.17)
	>40 years old	110/3188	1.07 (0.88-1.31)
Colorectum	None	900/40135	1.00 (ref)
	≤30 years old	361/14746	1.11 (0.98-1.25)
	31-40 years old	60/3003	1.00 (0.77-1.31)
	>40 years old	82/3188	1.17 (0.93-1.48)
Liver	None	388/40135	1.00 (ref)
	≤30 years old	136/14746	0.91 (0.75-1.11)
	31-40 years old	30/3003	1.04 (0.71-1.51)
	>40 years old	38/3188	1.13 (0.80-1.58)
Stomach	None	581/40135	1.00 (ref)
	≤30 years old	234/14746	1.07 (0.91-1.24)
	31-40 years old	50/3003	1.25 (0.94-1.68)
	>40 years old	37/3188	0.76 (0.54-1.06)
Thyroid	None	96/40135	1.00 (ref)
	≤30 years old	33/14746	1.00 (0.67-1.50)
	31-40 years old	5/3003	0.70 (0.28-1.72)

	>40 years old	5/3188	0.76 (0.31-1.90)
Esophagus	None	121/40135	1.00 (ref)
	≤30 years old	47/14746	0.87 (0.62-1.23)
	31-40 years old	8/3003	0.76 (0.37-1.56)
	>40 years old	15/3188	1.10 (0.64-1.90)
Prostate[b]	None	618/39949	1.00 (ref)
	≤30 years old	184/14680	0.90 (0.76-1.06)
	31-40 years old	24/2994	0.69 (0.46-1.04)
	>40 years old	38/3172	1.02 (0.73-1.42)
Bladder	None	224/40135	1.00 (ref)
	≤30 years old	77/14746	0.97 (0.74-1.26)
	31-40 years old	14/3003	0.96 (0.56-1.65)
	>40 years old	11/3188	0.67 (0.36-1.23)
Pancreas	None	229/40135	1.00 (ref)
	≤30 years old	98/14746	1.12 (0.88-1.44)
	31-40 years old	11/3003	0.74 (0.40-1.36)
	>40 years old	14/3188	0.81 (0.47-1.39)
Kidney	None	235/40135	1.00 (ref)
	≤30 years old	59/14746	0.73 (0.55-0.98)
	31-40 years old	18/3003	1.16 (0.71-1.87)
	>40 years old	10/3188	0.59 (0.31-1.12)

a. Adjusted for age at baseline, education, income, smoking, alcohol use, CHFP score, physical activity and BMI.
b. An additional 277 participants with a history of prostatectomy at baseline were excluded.

Table S6 Associations between cumulative duration of night shift work and cancer risk in men with more accurate adjustment for smoking and alcohol use

	Cumulative duration of night shift work	No. of cases/No. of participants	HR (95%CI) [a]	P_{trend} [b]	$P_{nonlinear}$ [c]
All cancers	None	5375/40135	1.00 (ref)	0.402	0.6083
	≤10 years	1827/13879	1.01 (0.95-1.06)		
	11-20 years	510/3959	1.01 (0.92-1.11)		
	>20 years	490/3099	1.04 (0.95-1.14)		
Major cancer site					
Lung	None	1092/40135	1.00 (ref)	0.5674	0.6969
	≤10 years	361/11493	1.05 (0.94-1.17)		
	11-20 years	116/3959	1.02 (0.84-1.24)		
	>20 years	112/3099	1.06 (0.87-1.30)		
Colorectum	None	900/40135	1.00 (ref)	0.1063	0.3405
	≤10 years	292/11493	1.09 (0.96-1.24)		
	11-20 years	84/3959	1.03 (0.82-1.29)		
	>20 years	94/3099	1.22 (0.98-1.52)		
Liver	None	388/40135	1.00 (ref)	0.6038	0.7515
	≤10 years	104/11493	0.97 (0.79-1.18)		
	11-20 years	37/3959	0.94 (0.67-1.32)		
	>20 years	34/3099	0.98 (0.69-1.41)		
Stomach	None	581/40135	1.00 (ref)	0.568	0.7529
	≤10 years	191/11493	1.06 (0.90-1.24)		
	11-20 years	63/3959	1.14 (0.88-1.48)		
	>20 years	48/3099	0.89 (0.66-1.20)		
Thyroid	None	96/40135	1.00 (ref)	0.6745	0.266
	≤10 years	25/11493	0.91 (0.60-1.38)		
	11-20 years	5/3959	0.56 (0.23-1.37)		

	>20 years	9/3099	1.62 (0.80-3.25)		
Esophagus	None	121/40135	1.00 (ref)	0.301	0.1448
	≤10 years	28/11493	0.78 (0.54-1.12)		
	11-20 years	12/3959	0.83 (0.46-1.51)		
	>20 years	20/3099	1.40 (0.86-2.28)		
Prostated	None	618/39949	1.00 (ref)	0.4313	0.9342
	≤10 years	163/13820	0.89 (0.75-1.06)		
	11-20 years	36/3943	0.78 (0.56-1.10)		
	>20 years	47/3083	0.96 (0.71-1.30)		
Bladder	None	224/40135	1.00 (ref)	0.3487	0.6238
	≤10 years	60/11493	0.94 (0.71-1.23)		
	11-20 years	20/3959	1.01 (0.64-1.61)		
	>20 years	14/3099	0.77 (0.44-1.32)		
Pancreas	None	229/40135	1.00 (ref)	0.7441	0.1411
	≤10 years	64/11493	0.94 (0.72-1.24)		
	11-20 years	32/3959	1.59 (1.09-2.31)		
	>20 years	15/3099	0.77 (0.46-1.31)		
Kidney	None	235/40135	1.00 (ref)	0.6651	0.8635
	≤10 years	50/11493	0.74 (0.55-1.00)		
	11-20 years	14/3959	0.68 (0.39-1.17)		
	>20 years	17/3099	1.02 (0.62-1.68)		

a. Adjusted for age at baseline, education, income, smoking, alcohol use, CHFP score, physical activity and BMI.
b. Modelled as a continuous variable to test for linear trend.
c. Nonlinear associations were tested among participants with a history of night shift work.
d. An additional 277 participants with a history of prostatectomy at baseline were excluded.

Table S7 Associations between frequency of night shifts and cancer risk in men with more accurate adjustment for smoking and alcohol use

	Cumulative duration of night shift work	No. of cases/No. of participants	HR (95%CI) [a]	P_{trend} [b]	$P_{nonlinear}$ [c]
All cancers	None	5375/40135	1.00 (ref)		
	≤8 nights/month	1900/14390	0.98 (0.93, 1.04)	0.2522	0.5089
	>8 nights/month	927/6548	1.06 (0.99, 1.14)		
Major cancer site					
Lung	None	1092/40135	1.00 (ref)		
	≤8 nights/month	422/14390	1.00 (0.89, 1.12)	0.0798	0.0602
	>8 nights/month	223/6548	1.14 (0.98, 1.31)		
Colorectum	None	900/40135	1.00 (ref)		
	≤8 nights/month	340/14390	1.08 (0.95, 1.23)	0.1526	0.9845
	>8 nights/month	163/6548	1.15 (0.97, 1.37)		
Liver	None	388/40135	1.00 (ref)		
	≤8 nights/month	136/14390	0.93 (0.77, 1.14)	0.7409	0.3739
	>8 nights/month	68/6548	1.03 (0.79, 1.33)		
Stomach	None	581/40135	1.00 (ref)		
	≤8 nights/month	214/14390	1.01 (0.86, 1.18)	0.3592	0.1091
	>8 nights/month	107/6548	1.12 (0.91, 1.38)		
Thyroid	None	96/40135	1.00 (ref)		
	≤8 nights/month	27/14390	0.85 (0.55, 1.31)	0.9827	0.4992
	>8 nights/month	16/6548	1.08 (0.63, 1.84)		
Esophagus	None	121/40135	1.00 (ref)		
	≤8 nights/month	43/14390	0.82 (0.57, 1.16)	0.7977	0.5329
	>8 nights/month	27/6548	1.07 (0.70, 1.63)		
Prostate[d]	None	618/39949	1.00 (ref)		
	≤8 nights/month	163/14318	0.84 (0.70, 1.00)	0.1514	0.9132
	>8 nights/month	83/6529	1.00 (0.79, 1.26)		
Bladder	None	224/40135	1.00 (ref)		
	≤8 nights/month	70/14390	0.91 (0.70, 1.20)	0.9944	0.2766
	>8 nights/month	32/6548	0.94 (0.65, 1.37)		
Pancreas	None	229/40135	1.00 (ref)		
	≤8 nights/month	81/14390	1.03 (0.79, 1.33)	0.8673	0.2356
	>8 nights/month	36/6548	1.03 (0.72, 1.46)		
Kidney	None	235/40135	1.00 (ref)		
	≤8 nights/month	61/14390	0.79 (0.59, 1.05)	0.1026	0.2262
	>8 nights/month	26/6548	0.73 (0.49, 1.10)		

a. Adjusted for age at baseline, education, income, smoking, alcohol use, CHFP score, physical activity and BMI.

b. Modeled as a continuous variable to test for linear trend.

c. Nonlinear associations were tested among participants with a history of night shift work.

d. An additional 277 participants with a history of prostatectomy at baseline were excluded.

Table S8 Associations between age at starting night shift work and cancer risk by cessation of night shift work

		Night shift work					
		Current workers		Recent quitters		Long-term quitters	
	Age at starting night shift work	No. of cases/No. of participants	HR (95%CI) [a]	No. of cases/No. of participants	HR (95%CI) [a]	No. of cases/No. of participants	HR (95%CI) [a]
All cancers	None	5375/40135	1.00 (ref)	5375/40135	1.00 (ref)	5375/40135	1.00 (ref)
	≤30 years old	120/1233	1.09 (0.91-1.31)	262/2273	1.03 (0.91-1.17)	1640/11242	1.01 (0.95-1.07)
	31-40 years old	88/954	1.12 (0.90-1.39)	104/1022	0.99 (0.82-1.21)	175/1027	0.96 (0.83-1.12)
	>40 years old	180/1714	0.96 (0.82-1.11)	179/1161	1.01 (0.87-1.18)	79/313	1.11 (0.89-1.39)
Major cancer site							
Lung	None	1092/40135	1.00 (ref)	1092/40135	1.00 (ref)	1092/40135	1.00 (ref)
	≤30 years old	26/1233	1.02 (0.69-1.51)	70/2273	1.19 (0.93-1.52)	362/11242	1.04 (0.93-1.18)
	31-40 years old	19/954	1.03 (0.65-1.62)	26/1022	1.05 (0.71-1.55)	32/1027	0.83 (0.58-1.18)
	>40 years old	46/1714	1.03 (0.76-1.39)	46/1161	1.10 (0.82-1.48)	18/313	1.13 (0.70-1.80)
Colorectum	None	900/40135	1.00 (ref)	900/40135	1.00 (ref)	900/40135	1.00 (ref)
	≤30 years old	16/1233	0.92 (0.55-1.51)	45/2273	1.11 (0.82-1.51)	300/11242	1.12 (0.98-1.28)
	31-40 years old	14/954	1.13 (0.66-1.93)	14/1022	0.85 (0.50-1.45)	32/1027	1.05 (0.73-1.49)
	>40 years old	29/1714	0.98 (0.67-1.42)	33/1161	1.18 (0.83-1.67)	20/313	1.73 (1.10-2.71)
Liver	None	388/40135	1.00 (ref)	388/40135	1.00 (ref)	388/40135	1.00 (ref)
	≤30 years old	13/1233	1.37 (0.78-2.41)	21/2273	1.00 (0.64-1.56)	102/11242	0.87 (0.70-1.08)
	31-40 years old	13/954	1.79 (1.02-3.16)	8/1022	0.91 (0.45-1.85)	9/1027	0.74 (0.38-1.43)
	>40 years old	20/1714	1.29 (0.82-2.04)	14/1161	1.09 (0.64-1.87)	4/313	0.85 (0.32-2.30)
Stomach	None	581/40135	1.00 (ref)	581/40135	1.00 (ref)	581/40135	1.00 (ref)
	≤30 years old	7/1233	0.60 (0.28-1.27)	26/2273	0.94 (0.63-1.40)	201/11242	1.11 (0.95-1.31)
	31-40 years old	11/954	1.34 (0.73-2.46)	12/1022	1.06 (0.60-1.90)	27/1027	1.30 (0.88-1.91)
	>40 years old	14/1714	0.67 (0.39-1.15)	14/1161	0.69 (0.41-1.18)	9/313	1.09 (0.56-2.11)
Thyroid	None	96/40135	1.00 (ref)	96/40135	1.00 (ref)	96/40135	1.00 (ref)
	≤30 years old	4/1233	1.20 (0.43-3.31)	2/2273	0.35 (0.09-1.44)	27/11242	1.12 (0.73-1.73)

	31-40 years old	2/954	0.73 (0.18-2.98)	3/1022	1.12 (0.35-3.57)	0/1027	–
	>40 years old	3/1714	0.73 (0.23-2.35)	2/1161	0.84 (0.21-3.46)	0/313	–
Esophagus	None	121/40135	1.00 (ref)	121/40135	1.00 (ref)	121/40135	1.00 (ref)
	≤30 years old	6/1233	1.75 (0.75-4.06)	11/2273	1.38 (0.74-2.58)	30/11242	0.70 (0.46-1.04)
	31-40 years old	1/954	0.41 (0.06-3.00)	4/1022	1.18 (0.43-3.23)	3/1027	0.61 (0.19-1.92)
	>40 years old	9/1714	1.49 (0.75-2.99)	4/1161	0.73 (0.27-2.00)	2/313	0.82 (0.20-3.36)
Prostate[b]	None	618/39949	1.00 (ref)	618/39949	1.00 (ref)	618/39949	1.00 (ref)
	≤30 years old	5/1233	0.72 (0.30-1.75)	13/2265	0.65 (0.37-1.14)	166/11184	0.93 (0.78-1.10)
	31-40 years old	1/954	0.22 (0.03-1.57)	3/1021	0.41 (0.13-1.29)	20/1019	0.86 (0.55-1.34)
	>40 years old	10/1713	0.78 (0.41-1.47)	19/1159	1.15 (0.72-1.82)	9/300	1.02 (0.52-1.97)
Bladder	None	224/40135	1.00 (ref)	224/40135	1.00 (ref)	224/40135	1.00 (ref)
	≤30 years old	7/1233	1.85 (0.85-4.02)	9/2273	0.98 (0.50-1.92)	61/11242	0.92 (0.69-1.23)
	31-40 years old	4/954	1.46 (0.53-4.02)	5/1022	1.35 (0.55-3.31)	5/1027	0.63 (0.26-1.54)
	>40 years old	4/1714	0.62 (0.23-1.68)	3/1161	0.44 (0.14-1.38)	4/313	1.36 (0.50-3.70)
Pancreas	None	229/40135	1.00 (ref)	229/40135	1.00 (ref)	229/40135	1.00 (ref)
	≤30 years old	7/1233	1.71 (0.79-3.71)	5/2273	0.51 (0.21-1.24)	80/11242	1.18 (0.92-1.53)
	31-40 years old	0/954	–	5/1022	1.27 (0.52-3.11)	6/1027	0.77 (0.34-1.74)
	>40 years old	6/1714	0.85 (0.37-1.94)	5/1161	0.72 (0.29-1.75)	3/313	1.01 (0.32-3.18)
Kidney	None	235/40135	1.00 (ref)	235/40135	1.00 (ref)	235/40135	1.00 (ref)
	≤30 years old	4/1233	0.69 (0.25-1.87)	8/2273	0.71 (0.35-1.44)	47/11242	0.75 (0.55-1.03)
	31-40 years old	5/954	1.14 (0.46-2.80)	4/1022	0.81 (0.30-2.18)	9/1027	1.49 (0.76-2.91)
	>40 years old	4/1714	0.47 (0.17-1.28)	5/1161	0.80 (0.33-1.95)	1/313	0.59 (0.08-4.23)

a. Adjusted for age at baseline, education, income, smoking, alcohol use, CHFP score, physical activity and BMI.
b. An additional 277 participants with a history of prostatectomy at baseline were excluded.
Hazard ratios and 95% confidence intervals that could not be estimated due to sparse data (very few events) in this category were presented as dash.

Table S9 Associations between cumulative duration of night shift work and cancer risk by cessation of night shift work

		Night shift work					
		Current workers		Recent quitters		Long-term quitters	
	Cumulative duration of night shift work	No. of cases/No. of participants	HR (95%CI) [a]	No. of cases/No. of participants	HR (95%CI) [a]	No. of cases/No. of participants	HR (95%CI) [a]
All cancers	None	5375/40135	1.00 (ref)	5375/40135	1.00 (ref)	5375/40135	1.00 (ref)
	≤10 years	233/2387	0.99 (0.86-1.13)	244/2040	1.01 (0.89-1.15)	1350/9454	1.00 (0.95-1.07)
	11-20 years	68/649	1.16 (0.91-1.48)	113/1249	0.89 (0.74-1.08)	329/2061	1.04 (0.93-1.16)
	>20 years	87/865	1.06 (0.85-1.31)	188/1167	1.11 (0.96-1.29)	215/1067	0.97 (0.85-1.12)
Major cancer site							
Lung	None	1092/40135	1.00 (ref)	1092/40135	1.00 (ref)	1092/40135	1.00 (ref)
	≤10 years	37/1500	1.00 (0.76-1.32)	37/1373	1.03 (0.79-1.35)	208/6334	1.06 (0.94-1.21)
	11-20 years	19/887	1.02 (0.60-1.74)	22/667	1.11 (0.78-1.57)	94/3120	0.99 (0.77-1.26)
	>20 years	35/1514	1.10 (0.71-1.70)	83/2416	1.30 (0.98-1.74)	110/3128	0.84 (0.61-1.16)
Colorectum	None	900/40135	1.00 (ref)	900/40135	1.00 (ref)	900/40135	1.00 (ref)
	≤10 years	19/1500	0.89 (0.62-1.27)	26/1373	1.00 (0.72-1.39)	167/6334	1.15 (1.00-1.32)
	11-20 years	14/887	1.42 (0.81-2.46)	12/667	1.02 (0.65-1.59)	87/3120	0.98 (0.74-1.30)
	>20 years	26/1514	0.98 (0.56-1.71)	54/2416	1.24 (0.88-1.75)	98/3128	1.32 (0.97-1.78)
Liver	None	388/40135	1.00 (ref)	388/40135	1.00 (ref)	388/40135	1.00 (ref)
	≤10 years	16/1500	1.44 (0.97-2.12)	14/1373	1.20 (0.78-1.83)	51/6334	0.84 (0.66-1.07)
	11-20 years	13/887	2.51 (1.43-4.40)	9/667	0.46 (0.19-1.11)	30/3120	0.84 (0.53-1.33)
	>20 years	17/1514	0.58 (0.22-1.56)	20/2416	1.20 (0.71-2.01)	34/3128	1.01 (0.59-1.71)
Stomach	None	581/40135	1.00 (ref)	581/40135	1.00 (ref)	581/40135	1.00 (ref)
	≤10 years	12/1500	0.74 (0.46-1.18)	14/1373	0.83 (0.54-1.27)	121/6334	1.14 (0.96-1.36)
	11-20 years	7/887	0.95 (0.42-2.15)	8/667	0.81 (0.45-1.49)	48/3120	1.27 (0.94-1.72)
	>20 years	13/1514	0.79 (0.37-1.68)	30/2416	1.00 (0.63-1.58)	68/3128	0.84 (0.55-1.30)
Thyroid	None	96/40135	1.00 (ref)	96/40135	1.00 (ref)	96/40135	1.00 (ref)
	≤10 years	2/1500	0.65 (0.23-1.78)	4/1373	1.01 (0.41-2.50)	14/6334	0.96 (0.59-1.55)

	11-20 years	2/887	—	1/667	—	6/3120	1.27 (0.51-3.13)
	>20 years	5/1514	2.22 (0.89-5.56)	2/2416	0.82 (0.20-3.38)	7/3128	1.85 (0.44-7.89)
Esophagus	None	121/40135	1.00 (ref)	121/40135	1.00 (ref)	121/40135	1.00 (ref)
	≤10 years	9/1500	1.33 (0.68-2.58)	5/1373	0.79 (0.35-1.81)	15/6334	0.64 (0.41-1.01)
	11-20 years	1/887	—	1/667	1.48 (0.64-3.40)	7/3120	0.69 (0.30-1.57)
	>20 years	6/1514	2.36 (1.02-5.49)	13/2416	1.34 (0.62-2.90)	13/3128	0.98 (0.45-2.15)
Prostate[b]	None	618/39949	1.00 (ref)	618/39949	1.00 (ref)	618/39949	1.00 (ref)
	≤10 years	13/2386	0.86 (0.49-1.51)	21/2038	1.10 (0.71-1.71)	129/9398	0.87 (0.72-1.05)
	11-20 years	0/649	—	2/1246	0.24 (0.06-0.95)	34/2048	0.99 (0.70-1.41)
	>20 years	3/865	0.54 (0.17-1.69)	12/1161	0.74 (0.42-1.32)	32/1057	1.17 (0.81-1.68)
Bladder	None	224/40135	1.00 (ref)	224/40135	1.00 (ref)	224/40135	1.00 (ref)
	≤10 years	5/1500	0.98 (0.47-2.02)	4/1373	0.79 (0.37-1.68)	39/6334	0.96 (0.71-1.30)
	11-20 years	3/887	1.98 (0.73-5.40)	3/667	0.90 (0.33-2.45)	14/3120	0.91 (0.51-1.64)
	>20 years	7/1514	1.05 (0.33-3.31)	10/2416	0.93 (0.41-2.10)	17/3128	0.55 (0.23-1.36)
Pancreas	None	229/40135	1.00 (ref)	229/40135	1.00 (ref)	229/40135	1.00 (ref)
	≤10 years	4/1500	0.68 (0.30-1.56)	5/1373	0.75 (0.35-1.61)	35/6334	1.02 (0.76-1.37)
	11-20 years	2/887	1.37 (0.43-4.34)	2/667	1.06 (0.43-2.59)	22/3120	1.84 (1.20-2.81)
	>20 years	7/1514	1.29 (0.47-3.52)	8/2416	0.45 (0.14-1.41)	32/3128	0.86 (0.42-1.77)
Kidney	None	235/40135	1.00 (ref)	235/40135	1.00 (ref)	235/40135	1.00 (ref)
	≤10 years	4/1500	0.52 (0.23-1.19)	4/1373	0.89 (0.45-1.74)	30/6334	0.77 (0.55-1.08)
	11-20 years	2/887	0.66 (0.16-2.69)	5/667	0.50 (0.16-1.57)	11/3120	0.79 (0.41-1.54)
	>20 years	7/1514	1.19 (0.48-2.91)	8/2416	0.79 (0.32-1.92)	16/3128	1.24 (0.57-2.69)

a. Adjusted for age at baseline, education, income, smoking, alcohol use, CHFP score, physical activity and BMI.

b. An additional 277 participants with a history of prostatectomy at baseline were excluded.

Hazard ratios and 95% confidence intervals that could not be estimated due to sparse data (very few events) in this category were presented as dash.

Table S10 Associations between frequency of night shifts and cancer risk by cessation of night shift work

		Night shift work					
		Current workers		Recent quitters		Long-term quitters	
	Average monthly frequency of night shifts	No. of cases/No. of participants	HR (95%CI) [a]	No. of cases/No. of participants	HR (95%CI) [a]	No. of cases/No. of participants	HR (95%CI) [a]
All cancers	None	5375/40135	1.00 (ref)	5375/40135	1.00 (ref)	5375/40135	1.00 (ref)
	≤8 nights/month	211/2315	0.95 (0.82-1.09)	345/2885	0.99 (0.89-1.11)	1344/9196	0.99 (0.93-1.05)
	>8 nights/month	177/1589	1.13 (0.97-1.32)	200/1574	1.05 (0.91-1.21)	550/3387	1.05 (0.96-1.15)
Major cancer site							
Lung	None	1092/40135	1.00 (ref)	1092/40135	1.00 (ref)	1092/40135	1.00 (ref)
	≤8 nights/month	47/2315	0.90 (0.67-1.22)	90/2885	1.12 (0.90-1.40)	285/9196	0.99 (0.86-1.13)
	>8 nights/month	44/1589	1.18 (0.87-1.61)	52/1574	1.14 (0.86-1.51)	127/3387	1.13 (0.94-1.36)
Colorectum	None	900/40135	1.00 (ref)	900/40135	1.00 (ref)	900/40135	1.00 (ref)
	≤8 nights/month	34/2315	0.97 (0.68-1.38)	53/2885	0.96 (0.73-1.28)	253/9196	1.13 (0.98-1.30)
	>8 nights/month	25/1589	1.01 (0.67-1.51)	39/1574	1.30 (0.94-1.79)	99/3387	1.15 (0.93-1.42)
Liver	None	388/40135	1.00 (ref)	388/40135	1.00 (ref)	388/40135	1.00 (ref)
	≤8 nights/month	26/2315	1.37 (0.91-2.07)	28/2885	1.01 (0.68-1.49)	82/9196	0.84 (0.66-1.07)
	>8 nights/month	20/1589	1.49 (0.94-2.36)	15/1574	1.01 (0.60-1.69)	33/3387	0.90 (0.63-1.28)
Stomach	None	581/40135	1.00 (ref)	581/40135	1.00 (ref)	581/40135	1.00 (ref)
	≤8 nights/month	14/2315	0.58 (0.34-0.99)	31/2885	0.81 (0.56-1.17)	169/9196	1.12 (0.94-1.33)
	>8 nights/month	18/1589	1.06 (0.66-1.71)	21/1574	0.99 (0.64-1.54)	68/3387	1.16 (0.90-1.50)
Thyroid	None	96/40135	1.00 (ref)	96/40135	1.00 (ref)	96/40135	1.00 (ref)
	≤8 nights/month	4/2315	0.67 (0.24-1.84)	1/2885	0.14 (0.02-1.04)	22/9196	1.16 (0.73-1.84)
	>8 nights/month	5/1589	1.19 (0.48-2.97)	6/1574	1.58 (0.68-3.65)	5/3387	0.72 (0.29-1.76)
Esophagus	None	121/40135	1.00 (ref)	121/40135	1.00 (ref)	121/40135	1.00 (ref)
	≤8 nights/month	8/2315	1.15 (0.55-2.40)	10/2885	0.94 (0.49-1.80)	25/9196	0.70 (0.45-1.08)
	>8 nights/month	8/1589	1.61 (0.77-3.37)	9/1574	1.44 (0.72-2.85)	10/3387	0.68 (0.36-1.31)
Prostate[b]	None	618/39949	1.00 (ref)	618/39949	1.00 (ref)	618/39949	1.00 (ref)

	≤8 nights/month	9/2315	0.62 (0.32-1.21)	23/2876	0.80 (0.53-1.23)	131/9133	0.86 (0.71-1.04)
	>8 nights/month	7/1588	0.70 (0.33-1.50)	12/1572	0.78 (0.44-1.40)	64/3371	1.09 (0.84-1.41)
Bladder	None	224/40135	1.00 (ref)	224/40135	1.00 (ref)	224/40135	1.00 (ref)
	≤8 nights/month	7/2315	0.90 (0.42-1.94)	10/2885	0.78 (0.41-1.48)	53/9196	0.95 (0.70-1.29)
	>8 nights/month	8/1589	1.49 (0.72-3.08)	7/1574	1.00 (0.47-2.14)	17/3387	0.79 (0.48-1.30)
Pancreas	None	229/40135	1.00 (ref)	229/40135	1.00 (ref)	229/40135	1.00 (ref)
	≤8 nights/month	9/2315	1.09 (0.55-2.16)	10/2885	0.74 (0.39-1.41)	62/9196	1.10 (0.82-1.45)
	>8 nights/month	4/1589	0.68 (0.25-1.84)	5/1574	0.69 (0.28-1.67)	27/3387	1.25 (0.83-1.86)
Kidney	None	235/40135	1.00 (ref)	235/40135	1.00 (ref)	235/40135	1.00 (ref)
	≤8 nights/month	6/2315	0.55 (0.24-1.24)	12/2885	0.82 (0.46-1.48)	43/9196	0.84 (0.61-1.17)
	>8 nights/month	7/1589	0.91 (0.42-1.96)	5/1574	0.63 (0.26-1.53)	14/3387	0.72 (0.42-1.24)

a. Adjusted for age at baseline, education, income, smoking, alcohol use, CHFP score, physical activity and BMI.

b. An additional 277 participants with a history of prostatectomy at baseline were excluded.

Table S11 Associations between combinations of night shift work duration and frequency and cancer risk

	night shift work	No. of cases/No. of participants	HR (95%CI) [a]
All cancers	None	5375/40135	1.00 (ref)
	short-term, low intensity	1197/9234	0.98 (0.92-1.04)
	short-term, high intensity	630/4644	1.045 (0.96-1.14)
	long-term, low intensity	703/5154	0.99 (0.92-1.08)
	long-term, high intensity	297/1903	1.11 (0.98-1.25)
Major cancer site			
Lung	None	1092/40135	1.00 (ref)
	short-term, low intensity	260/9234	0.99 (0.86-1.13)
	short-term, high intensity	157/4644	1.17 (0.99-1.38)
	long-term, low intensity	162/5154	1.03 (0.87-1.22)
	long-term, high intensity	66/1903	1.06 (0.83-1.37)
Colorectum	None	900/40135	1.00 (ref)
	short-term, low intensity	212/9234	1.06 (0.92-1.24)
	short-term, high intensity	113/4644	1.16 (0.95-1.41)
	long-term, low intensity	128/5154	1.11 (0.92-1.35)
	long-term, high intensity	50/1903	1.15 (0.86-1.53)
Liver	None	388/40135	1.00 (ref)
	short-term, low intensity	84/9234	0.92 (0.72-1.16)
	short-term, high intensity	49/4644	1.06 (0.79-1.43)
	long-term, low intensity	52/5154	0.96 (0.72-1.29)
	long-term, high intensity	19/1903	0.95 (0.60-1.51)
Stomach	None	581/40135	1.00 (ref)
	short-term, low intensity	134/9234	1.01 (0.83-1.22)
	short-term, high intensity	76/4644	1.16 (0.91-1.48)
	long-term, low intensity	80/5154	1.01 (0.80-1.29)
	long-term, high intensity	31/1903	1.04 (0.72-1.49)
Thyroid	None	96/40135	1.00 (ref)
	short-term, low intensity	19/9234	0.90 (0.55-1.48)
	short-term, high intensity	10/4644	0.92 (0.48-1.77)
	long-term, low intensity	8/5154	0.75 (0.36-1.56)
	long-term, high intensity	6/1903	1.52 (0.66-3.49)
Esophagus	None	121/40135	1.00 (ref)
	short-term, low intensity	21/9234	0.65 (0.41-1.04)
	short-term, high intensity	17/4644	1.01 (0.61-1.68)
	long-term, low intensity	22/5154	1.08 (0.68-1.71)
	long-term, high intensity	10/1903	1.19 (0.62-2.28)
Prostate[b]	None	618/39949	1.00 (ref)
	short-term, low intensity	105/9189	0.98 (0.72-1.34)
	short-term, high intensity	58/4630	0.85 (0.53-1.34)
	long-term, low intensity	58/5127	0.80 (0.51-1.24)
	long-term, high intensity	25/1898	1.15 (0.64-2.07)
Bladder	None	224/40135	1.00 (ref)
	short-term, low intensity	48/9234	0.84 (0.68-1.04)
	short-term, high intensity	20/4644	1.01 (0.77-1.32)

	long-term, low intensity	22/5154	0.84 (0.64-1.10)
	long-term, high intensity	12/1903	0.98 (0.66-1.47)
Pancreas	None	229/40135	1.00 (ref)
	short-term, low intensity	49/9234	0.98 (0.72-1.34)
	short-term, high intensity	21/4644	0.86 (0.55-1.35)
	long-term, low intensity	32/5154	1.12 (0.77-1.63)
	long-term, high intensity	15/1903	1.40 (0.83-2.37)
Kidney	None	235/40135	1.00 (ref)
	short-term, low intensity	37/9234	0.74 (0.52-1.04)
	short-term, high intensity	19/4644	0.75 (0.47-1.20)
	long-term, low intensity	24/5154	0.89 (0.58-1.36)
	long-term, high intensity	7/1903	0.68 (0.32-1.46)

a. Adjusted for age at baseline, education, income, smoking, alcohol use, CHFP score, physical activity and BMI.
b. An additional 277 participants with a history of prostatectomy at baseline were excluded.

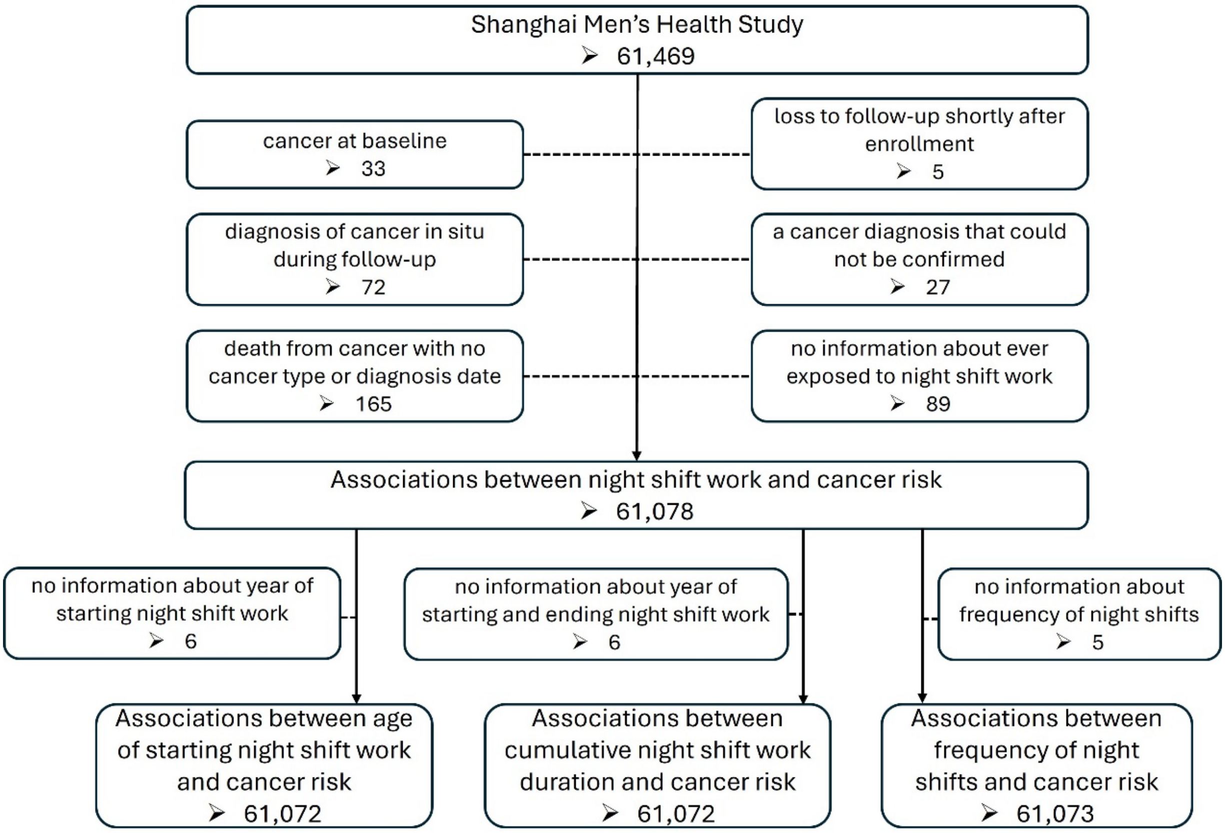

Figure S1 Flowchart of study participants

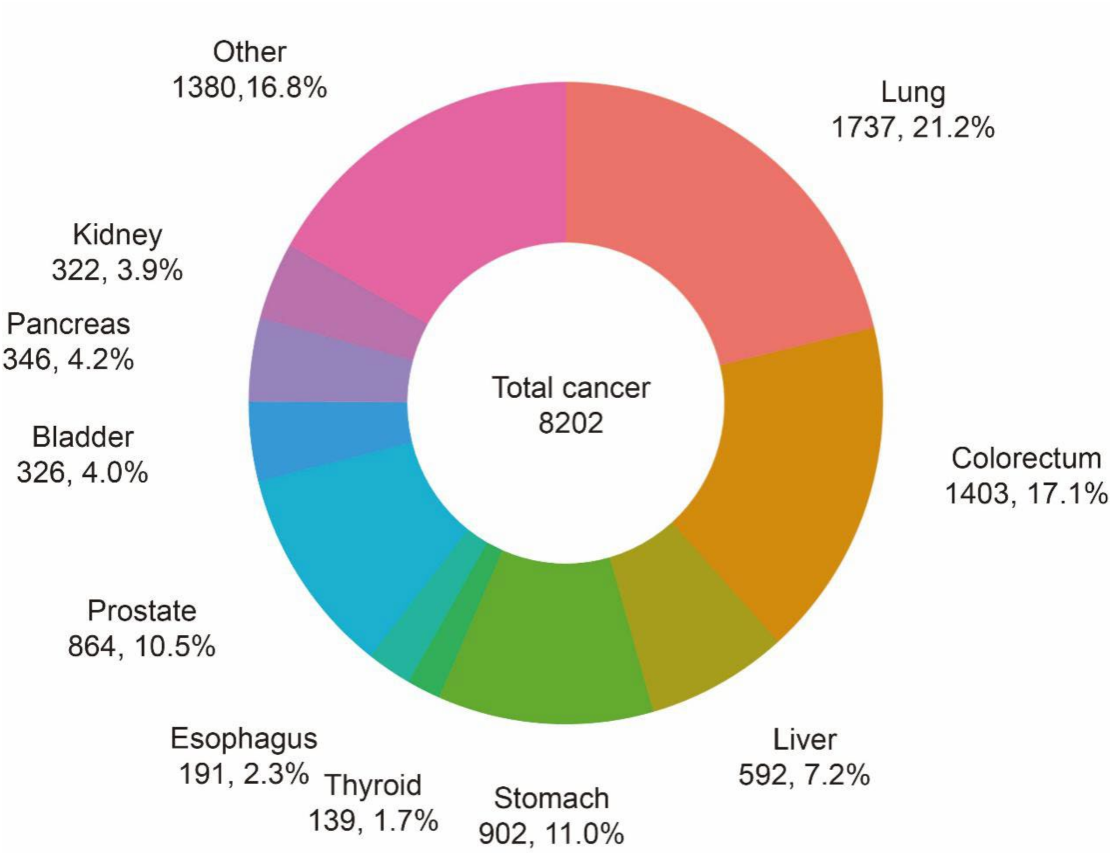

Figure S2 Count and proportion of incident cancer cases by 10 major sites

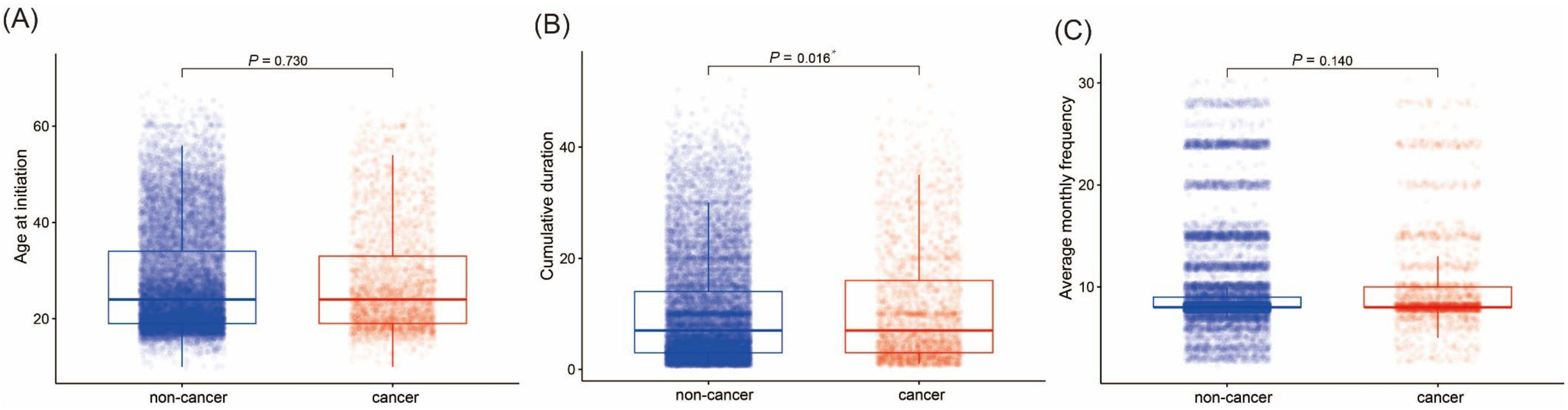

Figure S3 Distribution of night shift work metrics between cancer and non-cancer groups

(A) age at starting night shift work; (B) cumulative duration of night shift work; (C) average monthly frequency of night shifts
P values were obtained using the Mann-Whitney U test, with statistical significance indicated by an asterisk.

Cumulative duration	Cases/all	HR(95%CI)	$P_{interaction}$
**Stratified by smoking**			0.9984
Never smoking			
None	84/13147	1.00 (ref)	
≤10 years night work	21/3590	0.95 (0.59, 1.54)	
11−20 years night work	8/898	1.55 (0.74, 3.23)	
>20 years night work	5/905	0.80 (0.32, 2.02)	
Ever smoking			
None	145/26988	1.00 (ref)	
≤10 years night work	49/10289	0.94 (0.68, 1.31)	
11−20 years night work	24/3061	1.60 (1.04, 2.48)	
>20 years night work	10/2194	0.77 (0.40, 1.48)	
**Stratified by alcohol use**			0.9261
Never alcohol use			
None	166/27430	1.00 (ref)	
≤10 years night work	44/8704	0.89 (0.63, 1.24)	
11−20 years night work	20/2438	1.53 (0.96, 2.45)	
>20 years night work	10/1914	0.75 (0.39, 1.44)	
Ever alcohol use			
None	63/12705	1.00 (ref)	
≤10 years night work	26/5175	1.06 (0.67, 1.69)	
11−20 years night work	12/1521	1.72 (0.92, 3.22)	
>20 years night work	5/1185	0.83 (0.33, 2.07)	
**Stratified by diet**			0.2904
Diet score ≤ median			
None	112/19058	1.00 (ref)	
≤10 years night work	35/7396	0.88 (0.60, 1.29)	
11−20 years night work	17/2211	1.51 (0.90, 2.54)	
>20 years night work	8/1871	0.68 (0.33, 1.41)	
Diet score > median			
None	117/21077	1.00 (ref)	
≤10 years night work	35/6483	1.01 (0.69, 1.47)	
11−20 years night work	15/1748	1.65 (0.96, 2.85)	
>20 years night work	7/1228	0.89 (0.41, 1.93)	
**Stratified by physical activity**			0.5891
Physical activity ≤ median			
None	115/21080	1.00 (ref)	
≤10 years night work	31/6540	0.90 (0.60, 1.34)	
11−20 years night work	10/1765	1.14 (0.60, 2.19)	
>20 years night work	3/1152	0.41 (0.13, 1.30)	
Physical activity > median			
None	114/19055	1.00 (ref)	
≤10 years night work	39/7339	0.98 (0.68, 1.41)	
11−20 years night work	22/2194	1.94 (1.22, 3.09)	
>20 years night work	12/1947	0.99 (0.54, 1.82)	
**Stratified by BMI**			0.1088
BMI <24 $kg \cdot m^{-2}$			
None	120/21796	1.00 (ref)	
≤10 years night work	32/7617	0.80 (0.54, 1.19)	
11−20 years night work	20/2184	1.82 (1.12, 2.94)	
>20 years night work	5/1557	0.51 (0.21, 1.27)	
BMI ≥ 24 $kg \cdot m^{-2}$			
None	109/18339	1.00 (ref)	
≤10 years night work	38/6262	1.11 (0.76, 1.61)	
11−20 years night work	12/1775	1.33 (0.73, 2.42)	
>20 years night work	10/1542	1.03 (0.53, 1.99)	

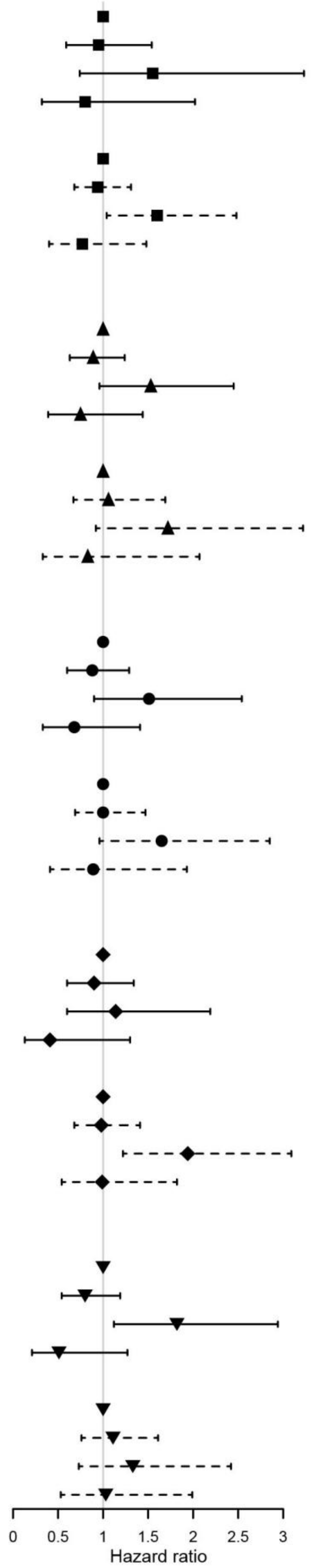

Figure S4 Stratified analysis of the association between cumulative duration of night shift work and pancreatic cancer risk according to lifestyle factors
